# Moderate intrinsic phenotypic alterations in *C9orf72* ALS/FTD iPSC-microglia despite the presence of C9orf72 pathological features

**DOI:** 10.3389/fncel.2023.1179796

**Published:** 2023-06-06

**Authors:** Ileana Lorenzini, Eric Alsop, Jennifer Levy, Lauren M. Gittings, Deepti Lall, Benjamin E. Rabichow, Stephen Moore, Ryan Pevey, Lynette M. Bustos, Camelia Burciu, Divya Bhatia, Mo Singer, Justin Saul, Amanda McQuade, Makis Tzioras, Thomas A. Mota, Amber Logemann, Jamie Rose, Sandra Almeida, Fen-Biao Gao, Michael Marks, Christopher J. Donnelly, Elizabeth Hutchins, Shu-Ting Hung, Justin Ichida, Robert Bowser, Tara Spires-Jones, Mathew Blurton-Jones, Tania F. Gendron, Robert H. Baloh, Kendall Van Keuren-Jensen, Rita Sattler

**Affiliations:** ^1^Department of Translational Neuroscience, Barrow Neurological Institute, Phoenix, AZ, United States; ^2^Neurogenomics Division, Translational Genomics Research Institute, Phoenix, AZ, United States; ^3^Center for Neural Science and Medicine, Cedars-Sinai Medical Center, Regenerative Medicine Institute, Los Angeles, CA, United States; ^4^School of Life Sciences, Arizona State University, Tempe, AZ, United States; ^5^Department of Neurobiology and Behavior, University of California, Irvine, Irvine, CA, United States; ^6^Sue and Bill Gross Stem Cell Research Center, University of California, Irvine, Irvine, CA, United States; ^7^Institute for Memory Impairments and Neurological Disorders, University of California, Irvine, Irvine, CA, United States; ^8^UK Dementia Research Institute, The University of Edinburgh, Edinburgh, United Kingdom; ^9^Centre for Brain Discovery Sciences, The University of Edinburgh, Edinburgh, United Kingdom; ^10^Department of Neurology, University of Massachusetts Medical School, Worcester, MA, United States; ^11^Department of Neurobiology, University of Pittsburgh School of Medicine, Pittsburgh, PA, United States; ^12^Department of Stem Cell Biology Regenerative Medicine, USC Keck School of Medicine, Los Angeles, CA, United States; ^13^Department of Neuroscience, Mayo Clinic, Jacksonville, FL, United States; ^14^Mayo Clinic Graduate School of Biomedical Sciences, Mayo Clinic, Jacksonville, FL, United States; ^15^Department of Neurology, Cedars-Sinai Medical Center, Los Angeles, CA, United States

**Keywords:** amyotrophic lateral sclerosis, C9orf72, frontotemporal dementia, iPSC-microglia, neuroinflammation

## Abstract

While motor and cortical neurons are affected in *C9orf72* amyotrophic lateral sclerosis and frontotemporal dementia (ALS/FTD), it remains largely unknown if and how non-neuronal cells induce or exacerbate neuronal damage. We differentiated *C9orf72* ALS/FTD patient-derived induced pluripotent stem cells into microglia (iPSC-MG) and examined their intrinsic phenotypes. Similar to iPSC motor neurons, *C9orf72* ALS/FTD iPSC-MG mono-cultures form G_4_C_2_ repeat RNA foci, exhibit reduced C9orf72 protein levels, and generate dipeptide repeat proteins. Healthy control and *C9orf72* ALS/FTD iPSC-MG equally express microglial specific genes and perform microglial functions, including inflammatory cytokine release and phagocytosis of extracellular cargos, such as synthetic amyloid beta peptides and healthy human brain synaptoneurosomes. RNA sequencing analysis revealed select transcriptional changes of genes associated with neuroinflammation or neurodegeneration in diseased microglia yet no significant differentially expressed microglial-enriched genes. Moderate molecular and functional differences were observed in *C9orf72* iPSC-MG mono-cultures despite the presence of C9orf72 pathological features suggesting that a diseased microenvironment may be required to induce phenotypic changes in microglial cells and the associated neuronal dysfunction seen in C9orf72 ALS/FTD neurodegeneration.

## Introduction

The GGGGCC (G_4_C_2_) hexanucleotide repeat expansion (HRE) in the non-coding region of the *chromosome 9 open reading frame 72* (*C9orf72)* gene is considered the most prevalent genetic abnormality associated with the spectrum disease of amyotrophic lateral sclerosis and frontotemporal dementia (ALS/FTD) to date (DeJesus-Hernandez et al., 2011; Renton et al., 2011b). The *C9orf72* HRE has been hypothesized to contribute to neurodegeneration through three non-mutually exclusive mechanisms. First, *C9orf72* HRE leads to haploinsufficiency of the C9orf72 protein expression due to a failure in the transcription of the expanded allele; second, non-canonical translation of the repeat RNA leads to the synthesis of dipeptide repeat (DPR) proteins; third, toxic RNA gain-of-function occurs via sequestration of RNA binding proteins to G_4_C_2_ RNA foci. Additionally, *C9orf72* postmortem tissues exhibit TAR-DNA binding protein 43 (TDP-43) pathology, characterized by nuclear depletion and cytoplasmic inclusions of TDP-43 (Neumann et al., 2006; Nakashima-Yasuda et al., 2007; Uryu et al., 2008; Lagier-Tourenne et al., 2010; Ling et al., 2013; Josephs et al., 2016).

While neuronal degeneration is a hallmark of ALS/FTD, it is well known that glia can impact the onset and progression of diseases via non-cell autonomous disease mechanisms (Barbeito et al., 2004; Yamanaka and Yamashita, 2007; Yamanaka et al., 2008; Ilieva et al., 2009; Haidet-Phillips et al., 2011; Kang et al., 2013; Brites and Vaz, 2014; Frakes et al., 2014; Madill et al., 2017; Zhao et al., 2020; Ghasemi et al., 2021). *C9orf72*, known to be differentially expressed in the central nervous system (CNS), is most highly expressed in peripheral myeloid cells and microglia (O'Rourke et al., 2016; Rizzu et al., 2016; Zhang et al., 2016a), stressing the need to understand the role and contribution of mutant *C9orf72* microglia to disease pathogenesis. *C9orf72* ALS/FTD postmortem brain tissues exhibit extensive microglial pathology in the corticospinal tract and corpus callosum compared to non-*C9orf72* ALS patients and control cases (Brettschneider et al., 2012; Cooper-Knock et al., 2012; Cardenas et al., 2017). Other reports showed enlarged CD68-positive lysosomes in microglia of the motor cortex and spinal cord of *C9orf72*-ALS patients compared to sporadic ALS (sALS), indicating active phagocytic activity in these regions (O'Rourke et al., 2016). With respect to *C9orf72* ALS/FTD cellular phenotypes, it is notable that pathological features examined in postmortem human tissues, such as the formation of nuclear RNA foci and the generation of DPR proteins, are most prominent in neurons compared to neighboring astrocytes and microglia (Gendron et al., 2013; Mackenzie et al., 2013; Mizielinska et al., 2013; DeJesus-Hernandez et al., 2017; Saberi et al., 2018; Rostalski et al., 2019).

While several *C9orf72* ALS/FTD mouse models exhibit gliosis and inflammation (Liu et al., 2016; O'Rourke et al., 2016; Schludi et al., 2017; Zhang et al., 2018), mouse models of *C9orf72* deficiency show a more defined contribution of microglia to disease phenotypes. *C9orf72* knockout mice display microglia lysosomal accumulation and increased expression of pro-inflammatory cytokines including IL6 and IL1β (Burberry et al., 2016; Jiang et al., 2016; O'Rourke et al., 2016). In addition, transcriptomic profiling revealed an upregulation of inflammatory pathways similar to what has been found in *C9orf72* FTD patient tissues (Prudencio et al., 2015; O'Rourke et al., 2016). Similarly, knocking out *C9orf72* with antisense oligonucleotides in mice led to the upregulation of *TREM2* and *C1qa*, which are both upregulated in activated microglia (Lagier-Tourenne et al., 2013). Furthermore, we recently showed that *C9orf72*-deficient mouse microglia change their transcriptional profile to an enhanced inflammatory type I IFN signature and promote microglia-mediated synapse loss, suggesting a direct contribution of these cells to neurodegeneration (Lall et al., 2021). While these *in vivo* mouse models indicate that loss of *C9orf72* in microglia displays non-cell autonomous regulatory activities, it is still unknown whether the presence of endogenous *C9orf72* HREs in patient-derived human microglia leads to inherent phenotypic changes, which in turn could contribute non-cell autonomously to *C9orf72* disease pathogenesis. Additionally, microglia responses and activation states are known to be unique to the disease environment, and, therefore, neuron-microglia communication may be required to regulate mutant *C9orf72* microglial activation and exacerbation of disease phenotypes during neurodegeneration in *C9orf72* ALS/FTD. This hypothesis is supported by recent studies using microglia single-cell expression analyses of different pathological conditions to show that, while there is a conserved gene core program, a spectrum of microglia responses and activation states are unique to the disease environment (Smith and Dragunow, 2014; Galatro et al., 2017; Gosselin et al., 2017; Friedman et al., 2018; Geirsdottir et al., 2019). Finally*, in vitro* studies on the intrinsic properties of *C9orf72* microglia were recently assessed by overexpressing *C9orf72* HRE in mouse BV-2 microglial cells (Rostalski et al., 2020). While BV-2 microglial cells are presented with *C9orf72-*associated phenotypes, such as the production of DPR proteins, the presence of *C9orf72* HRE in microglia did not alter their functions or viability.

To better understand the contribution of microglial cells to *C9orf72* ALS/FTD, we used an endogenous human *in vitro* cell culture model by differentiating mutant *C9orf72* ALS/FTD patient-derived induced pluripotent stem cells into microglia (iPSC-MG). All iPSC-MG mono-cultures express known microglial genes and proteins. iPSC-MG carrying the endogenous *C9orf72* HRE recapitulate pathological hallmarks of *C9orf72* ALS/FTD disease, such as the formation of HRE-associated RNA foci, expression of poly (GP) DPRs, and reduced C9orf72 protein levels. Interestingly, despite the presence of *C9orf72* pathobiology, iPSC-MG grown as mono-cultures show minor gene expression changes compared to control iPSC-MG. Similarly, mutant *C9orf72* ALS/FTD and control iPSC-MG equally perform common microglial functions, such as the release of cytokines and chemokines upon exposure to extracellular lipopolysaccharide (LPS). In addition, *C9orf72* ALS/FTD iPSC-MG were indistinguishable from healthy control iPSC-MG when performing cytochalasin-D-dependent phagocytic activity upon exposure to human healthy brain synaptoneurosomes and engulfment of amyloid beta (Aβ) (1–40)-TAMRA. Our data suggest that a diseased CNS microenvironment is required to induce disease-specific microglial phenotypic changes that have been described in postmortem patient brain tissues and animal models of disease.

## Results

### *C9orf72* ALS/FTD patient and control iPSCs differentiate into brain-like microglia

**Figure 1:**
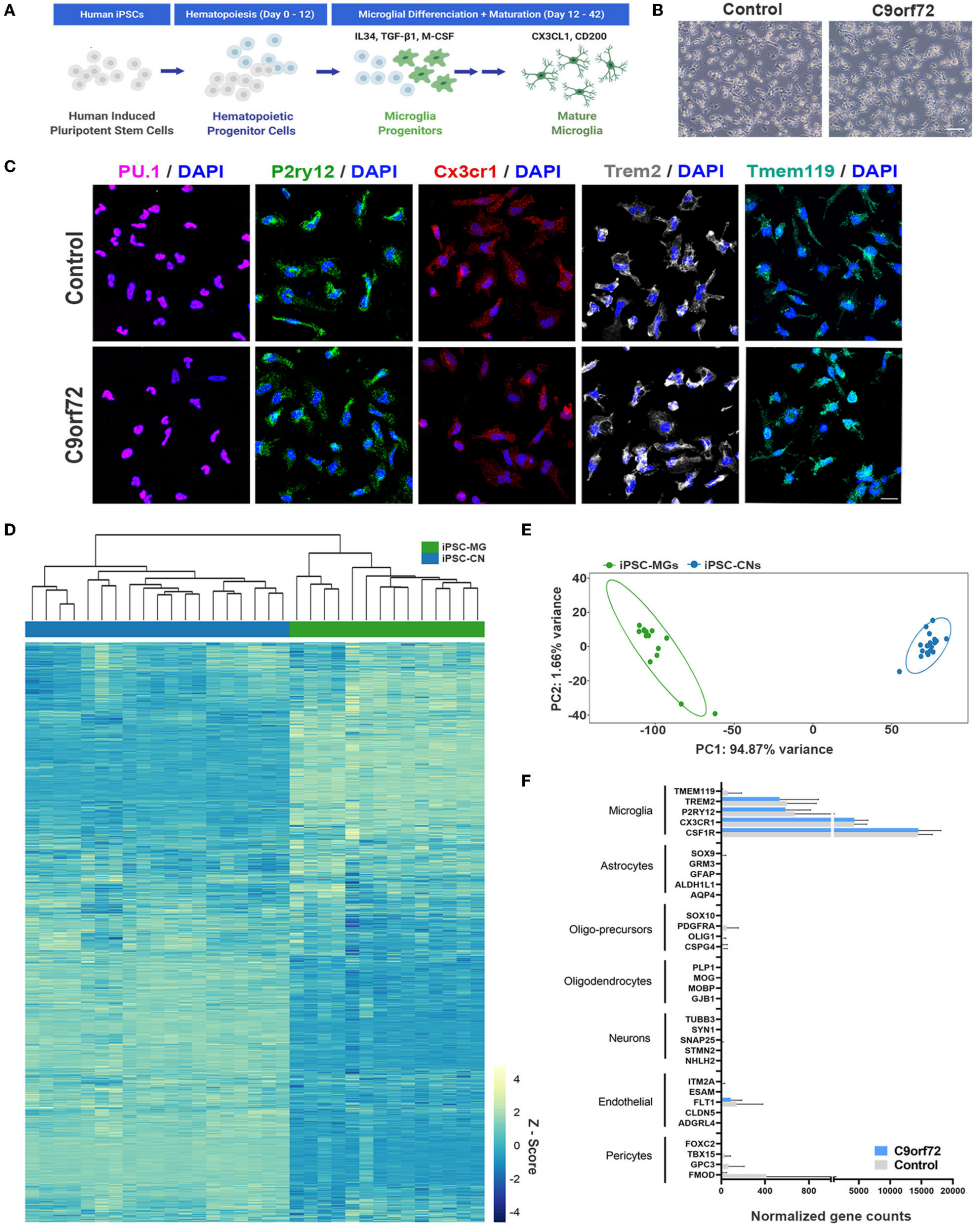
Healthy control and mutant *C9orf72* ALS/FTD patient iPSC lines differentiate into mature microglia. **(A)** Schematic illustration of iPSC-MG differentiation protocol. **(B)** Phase contrast images of mature iPSC-MG differentiated from healthy control and *C9orf72* ALS/FTD iPSCs (DIV 40). The representative images show typical ramified microglia morphology in both experimental groups. Scale bar, 120 μm. **(C)** Representative immunofluorescence of DIV 40 control (*n* = 3) and *C9orf72* ALS/FTD iPSC-MG (*n* = 3–5) stained for myeloid transcription factor PU.1 and microglia specific markers such as purinergic surface receptor P2ry12, C-X3-C Motif Chemokine Receptor 1 (Cx3cr1), triggering receptor expressed on myeloid cells 2 (TREM2), and the transmembrane protein 119 (TMEM119). For quantification of marker protein expression/percentage of DAPI-positive cells, see Supplementary Figure S1A. For quantitative marker gene expression levels, see Supplementary Figure S1B. Scale bar, 20 μm. **(D)** Heatmap of the iPSC-MG (control, *n* = 4 cell lines with 1–2 differentiations each and *C9orf72* ALS/FTD, *n* = 7 cell lines with 1–2 differentiations each) and iPSC-CN (*n* = 12 cell lines with 1-3 differentiations each) transcriptome demonstrating distinct gene expression profiles between the two cell populations. All iPSC-MG and iPSC-CN samples were normalized together by DESeq2 and Z-score scaled. **(E)** Principal component analysis of the RNA-seq expression data revealed a highly similar gene expression profile within both iPSC-MG (green cluster) and iPSC-CN (blue cluster) and confirmed these populations as distinct from each other (PC1, 94.87% variance; PC2, 1.66% variance). **(F)** Normalized counts for genes associated with microglia, astrocytes, oligo-precursor cells (OPC), oligodendrocytes, neurons, endothelial cells, and pericytes within the iPSC-MG population (Control *n* = 4 lines; *C9orf72* ALS/FTD *n* = 7 lines). Bar graphs are presented as mean ± SD.

Following established protocols (Abud et al., 2017; McQuade et al., 2018), we differentiated one isogenic pair, up to seven control and eight *C9orf72* ALS/FTD patient iPSC lines into microglia (Figure 1A, Supplementary Tables S1–3). At a mature stage of 40 days *in vitro* (DIV), both control and *C9orf72* ALS/FTD iPSC-MG display a typical ramified microglia morphology (Figure 1B) and express classic microglial marker proteins including the myeloid transcription factor PU.1, purinergic surface receptor P2RY12, and C-X3-C Motif Chemokine Receptor 1 (CX3CR1), as confirmed via fluorescent immunostaining (Figure 1C, Supplementary Figure S1A). Differentiated microglia uniformly expressed the triggering receptor expressed on myeloid cells 2 (TREM2) and transmembrane protein 119 (TMEM119), further demonstrating a commitment to microglial fate, with no differences detected between *C9orf72* and control iPSC-MG (Figure 1C, Supplementary Figures S1A, B). To validate microglial lineage, we performed Illumina paired-end deep RNA sequencing analysis (RNA seq) on four control and seven *C9orf72* ALS/FTD lines of iPSC-MG (Figures 1D, E). At a transcriptional level, control and *C9orf72* iPSC-MG clustered together and presented with a unique transcriptome compared to iPSCs differentiated into forebrain cortical neurons (iPSC-CN) (Figure 1D). Principal component analysis of this RNA seq dataset revealed a highly similar gene expression profile within iPSC-MG and showed a significant difference from iPSC-CNs confirming that these populations are distinct from each other (PC1, 94.87% variance; PC2, 1.66% variance; Figure 1E). To verify the absence of other cell types in the differentiated iPSC-MG cultures, we assessed the expression of known cell type-specific markers which revealed high expression of microglial-enriched genes and low expression of gene transcripts unique to astrocytes, oligodendrocyte precursor cells (OPC), oligodendrocytes, neurons, endothelial cells, and pericytes (Figure 1F) (Zhang et al., 2014). We observed the expression of endothelial and pericyte marker genes *FTL1, GPC3*, and *FMOD*; however, these genes are known to also be expressed in microglia (https://www.proteinatlas.org/). These data confirm that the differentiation of microglia from iPSC results in the production of brain-like microglia that express microglial-enriched genes and proteins, distinct from other cell types, yet no inherent differences were observed between control and human *C9orf72* ALS/FTD patient iPSC-MGs.

### *C9orf72* ALS/FTD iPSC-MG transcriptome profile shows moderate overall gene expression changes yet an unaltered expression of microglia-enriched genes

**Figure 2:**
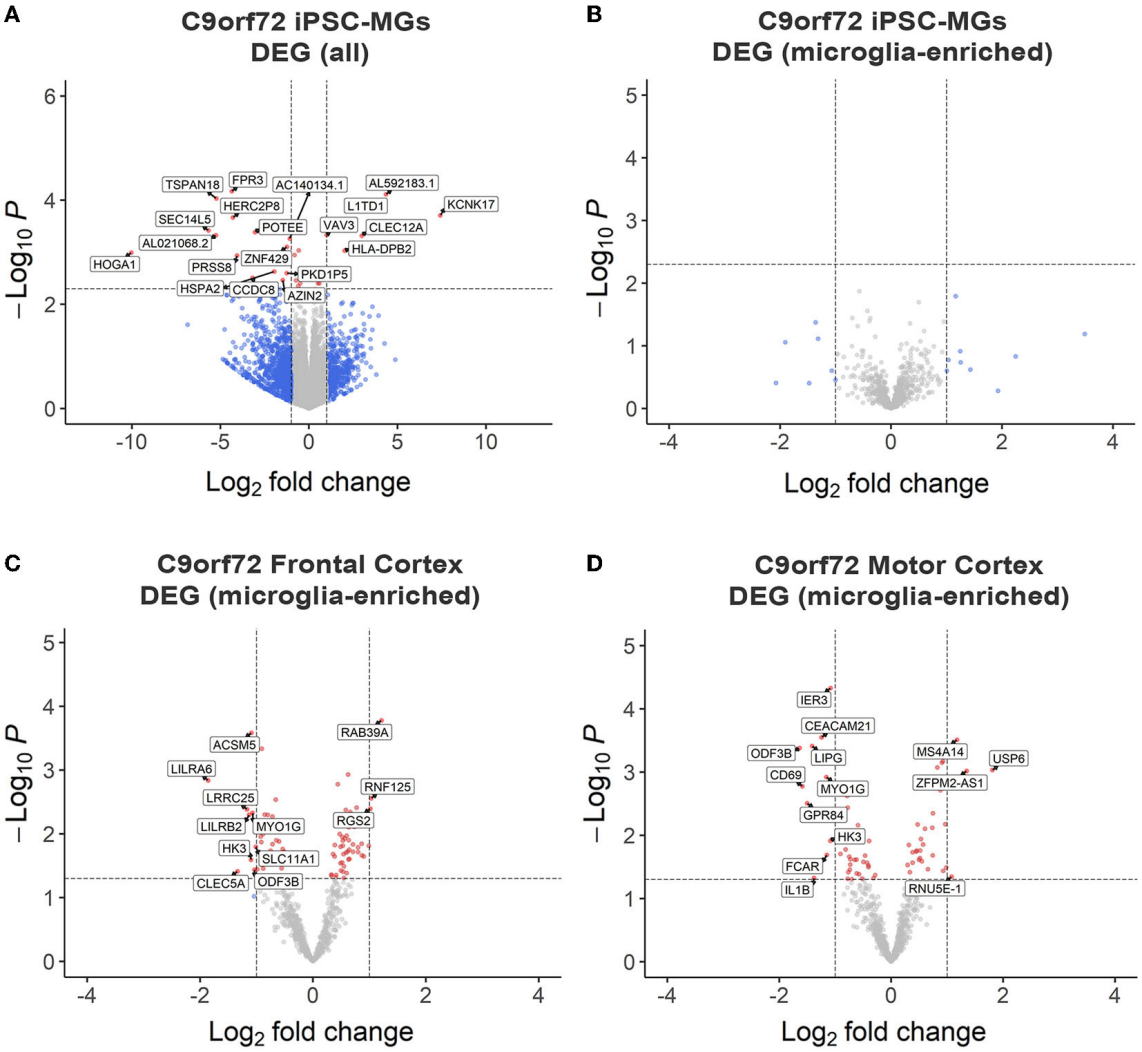
RNA sequencing analyses revealed minor transcriptional changes in mono-cultures of *C9orf72* ALS/FTD iPSC-MG and postmortem brain tissues of *C9orf72* ALS/FTD patients. **(A)** Volcano plot showing differentially expressed transcripts between healthy control (*n* = 4) and *C9orf72* ALS/FTD (*n* = 7) from the full iPSC-MG transcriptome (unadjusted p-value < 0.005; log_2_ fold change (FC) ± 1). Using these selection criteria, 20 genes were found to be differentially expressed in *C9orf72* ALS/FTD iPSC-MG. **(B)** Volcano plot of differentially expressed microglial-enriched transcripts (total of 881) indicates that there are no significant expression changes of these particular genes in mono-cultures of *C9orf72* ALS/FTD iPSC-MG (*n* = 7) compared to healthy control (*n* = 4) (unadjusted p-value <0.005; log_2_ fold change (FC) ± 1). **(C, D)** Differentially expressed microglia-enriched transcripts in postmortem brain tissues from the frontal cortex (control *n* = 16; *C9orf72* ALS/FTD *n* = 8) and motor cortex (control *n* = 15; *C9orf72* ALS/FTD *n* = 12) from the Target ALS dataset. Volcano plots show 12 differentially expressed microglia transcripts in *C9orf72* ALS/FTD for frontal cortex **(C)** and 14 for motor cortex **(D)** when compared to controls (unadjusted p-value <0.05; log_2_ fold change (FC) ± 1).

To test if *C9orf72* microglia exhibit intrinsic disease-mediated gene expression alterations, we analyzed our RNA sequencing data to determine differentially expressed genes (DEGs) between mature control and *C9orf72* ALS/FTD iPSC-MG. This analysis revealed a few DEGs with significantly altered expression in *C9orf72* ALS/FTD iPSC-MG [20 genes; log_2_ fold change (FC) ± 1, unadjusted *p* < 0.005 Figure 2A, Supplementary Figure S2A]. The topmost downregulated genes included *HOGA1, SEC14L5, FPR3*, TSPAN18, and SEC14L5. Among the topmost upregulate genes are *KCNK17, L1TD1, CLEC12A*, and *VAV3*. We next determined if 881 RNA transcripts previously reported to be enriched in cortical microglia were differentially expressed in our dataset but found no significant dysregulation of these select genes (Figure 2B) (Gosselin et al., 2017). Additionally, RNA sequencing data from iPSC-MG cells did not show differences in homeostatic, interferon, activated, or NF-κB response genes (Lall et al., 2021), although varied gene expression was observed among patient lines (Supplementary Figures S3A–D). These data suggest that the *C9orf72* HRE in iPSC-MG induces subtle changes in the overall cellular transcriptome including select genes associated with neuroinflammatory pathways. No significant alterations were detected in microglial-enriched genes when *C9orf72* iPCS-MG were grown in mono-cultures without surrounding CNS cell types.

To test this hypothesis, we analyzed existing RNA sequencing datasets obtained through the Target ALS consortium and the New York Genome Center. We evaluated gene expression changes of the 881 microglial-enriched RNA transcripts in the frontal cortex and motor cortex brain tissues of *C9orf72* ALS/FTD patients (Figures 2C, D, Supplementary Figure S2B, Supplementary Table 4). We also quantified gene expression changes in the occipital cortex as a brain region considered to be less affected by C9orf72 disease pathology (Supplementary Figures S2C, D, Supplementary Table S4). From the 881 microglial-enriched genes, 12 were dysregulated in the frontal cortex: *LILRA6, LRRCA, MYO1G, RAB39A, ACSM5, RNF125*, and *RGS2*; 14 microglial-enriched genes were dysregulated in the motor cortex (e.g., *CD69, CEACAM21, GPR84, LIPG*, and *MYO1G)* and 21 in the occipital cortex (e.g., *CD69, EGR2, GPR183, RGS1*, and *SPP1*; Figures 2C, D, Supplementary Figures S2B–D). The RNA sequencing data from *C9orf72* ALS/FTD human tissue, similar to *C9orf72* ALS/FTD iPSC-MG, did not show apparent differences in genes related to homeostatic, interferon, activated, or NF-κB pathways (Supplementary Figures S3E–H). The minimal changes observed in microglia-enriched genes from the bulk RNA seq datasets support the need for single-cell or single-nuclei RNA sequencing approaches to provide a more definite answer regarding microglia-specific differentially expressed genes in *C9orf72* disease.

### *C9orf72* ALS/FTD iPSC-MG have reduced C9orf72 protein levels and express *C9orf72* hexanucleotide repeat expansion-associated RNA foci and poly-(GP) DPR protein

**Figure 3:**
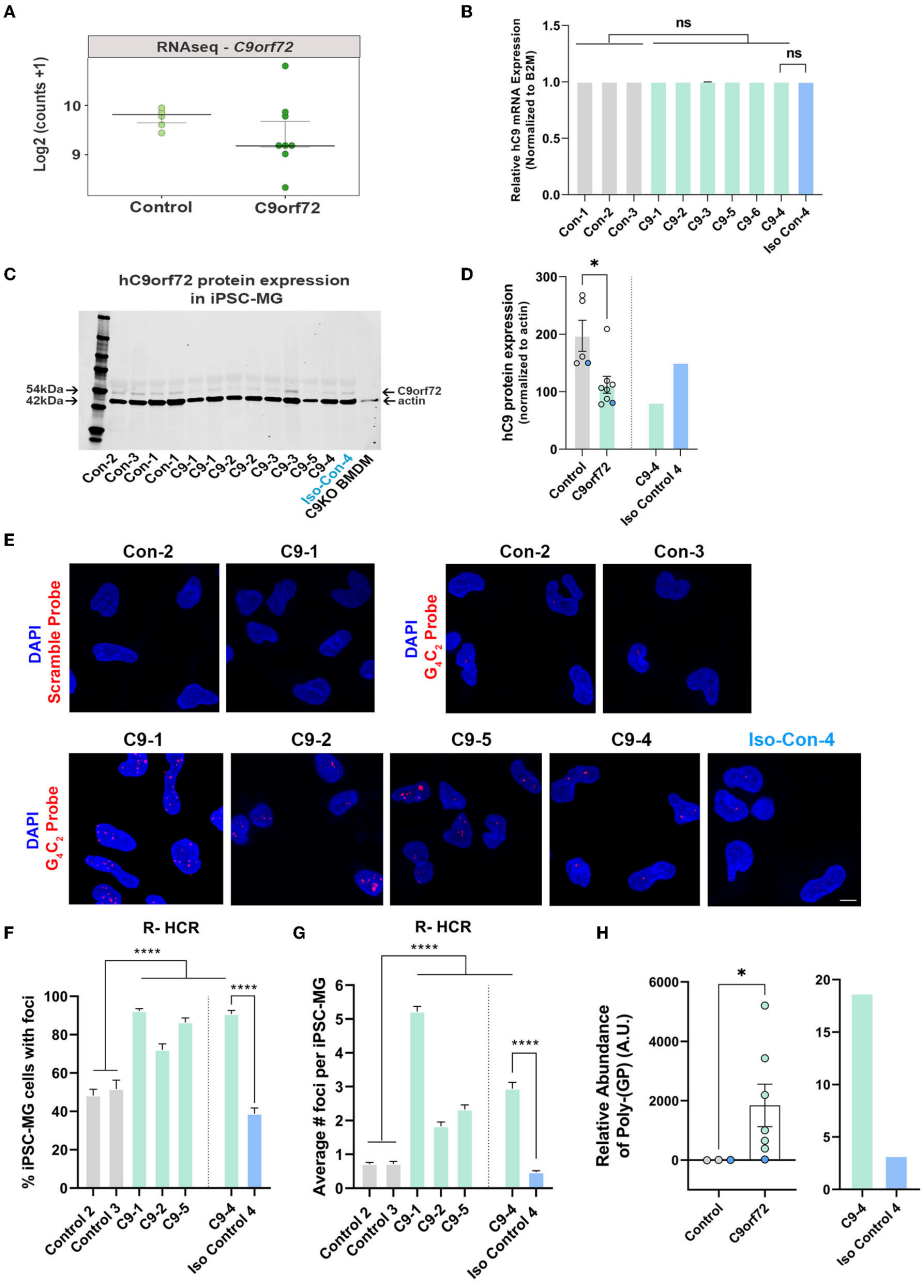
*C9orf72* ALS/FTD iPSC-MG exhibit reduced C9orf72 protein expression, present intranuclear HRE-associated RNA foci, and produce poly-(GP) DPR protein. **(A)** Dot plot showing *C9orf72* level of expression as log2 (counts +1) in control and *C9orf72* ALS/FTD iPSC-MG (Control, *n* = 4 lines; *C9orf72, n* = 7 lines; multiple differentiations per line are shown as individual data points; Student's *t*-test). **(B)** Relative human *C9orf72* mRNA expression performed by qRT-PCR in control and *C9orf72* ALS/FTD iPSC-MG (normalized expression to beta-2-microglobulin (B2M) in control, *n* = 3 lines, *n* = 1–4 differentiations per line; *C9orf72, n* = 6 lines, *n* = 1–3 differentiations per line; Student's t-test). **(C)** Western blot analysis shows a reduction in human C9orf72 protein expression in *C9orf72* ALS/FTD iPSC-MG. The 54 kDa C9orf72 protein band and 42 kDa actin protein band were used as a loading control (control, *n* = 4 lines, including an isogenic control-4, *n* = 1–2 differentiation per line; *C9orf72, n* = 5 lines, *n* = 1–2 differentiation per line). Bone marrow-derived macrophages (BMDM) from a *C9orf72* knockout mouse were used to validate the antibody used for the Western blot analysis. **(D)** Quantification of C9orf72 Western blot analysis revealed a significant reduction in C9orf72 protein levels (C9orf72 protein expression normalized to actin; control, 197.4, *n* = 4 lines, *n* = 1–2 differentiation per line; *C9orf72*, 112.1, *n* = 5 lines, *n* = 1–2 differentiation per line; p-value = 0.0115; Student's t-test, ^*^p ≤ 0.05). Blue dots in the bar graph represent C9-4 and its isogenic pair. **(E)** Representative images of control and *C9orf72* iPSC-MG treated with scramble or *C9orf72*-(G_4_C_2_)_6_ R-HCR initiator probes. Images show the presence of repeat-associated RNA foci in *C9orf72* ALS/FTD iPSC-MG. Scale bar = 20 μm. **(F)** Quantification of the percentage of iPSC-MG with detectable G_4_C_2_ RNA foci. A significant difference was observed for all C9 iPSC-MG lines when compared to control lines, including C9-4 as compared with corrected isogenic line 4 (control, *n* = 3 lines, including an isogenic control-4; *C9orf72, n* = 4 lines, one-way ANOVA followed by Tukey's multiple comparison test, ^****^p ≤ 0.0001). **(G)** Quantification of RNA foci number per iPSC-MG cell line. The evident increase in RNA foci number in C9 iPSG-MG when compared to control lines (control, *n* = 3 lines, including an isogenic control-4; *C9orf72, n* = 4 lines, one-way ANOVA followed by Tukey's multiple comparison test, ^****^p < 0.0001). **(H)** A significant increase in intracellular levels of poly-(GP) was detected in *C9orf72* ALS/FTD iPSC-MG using an ELISA assay (relative abundance of poly-(GP) in control, 5.46, *n* = 3 lines; *C9orf72*, 46.18, *n* = 7 lines, *p* = 0.0167, two-tailed Mann–Whitney test). Data are presented as Median ± SEM for *C9orf72* level expression. Exact significant p-values are reported in the figure legend. ^****^p ≤ 0.0001, ^*^p ≤ 0.05.

Glial cells have been shown to exhibit *C9orf72*-associated phenotypes in postmortem tissue, including HRE-associated RNA foci and DPR proteins, albeit to a lesser extent than neurons (Ash et al., 2013; Lagier-Tourenne et al., 2013; Mackenzie et al., 2013; Mizielinska et al., 2013; Sareen et al., 2013; Zhao et al., 2020). To evaluate *C9orf72* iPSC-MG for *C9orf72*-specific disease mechanisms, we first tested *C9orf72* iPSC-MGs for haploinsufficiency by examining the levels of the *C9orf72* transcript in *C9orf72* ALS/FTD and control iPSC-MG. Many studies have reported *C9orf72* haploinsufficiency in patient tissue; however, variable results have been observed in human patient-derived *C9orf72* iPSC-differentiated cells, including neurons and astrocytes (Donnelly et al., 2013; Sareen et al., 2013; Zhao et al., 2020). No significant differences in *C9orf72* transcript levels were detected between control and *C9orf72* groups in our RNA seq dataset (Figure 3A) or by quantitative RT-PCR of *C9orf72* (Figure 3B), which is consistent with previously published data (Sareen et al., 2013; Zhao et al., 2020). To measure C9orf72 protein levels, we performed quantitative Western blot analysis from control and *C9orf72* ALS/FTD iPSC-MG lysates, which revealed a significant reduction of C9orf72 protein in *C9orf72* ALS/FTD iPSC-MGs and was rescued in an isogenic control line (Figures 3C, D).

We then performed a repeat-hybridization chain reaction (R-HCR) in mature *C9orf72* iPSC-MG, a more sensitive approach to detect the presence of intranuclear RNA foci (Glineburg et al., 2021) (Figure 3E). A high percentage of mutant *C9orf72* iPSC-MG contained repeat associated intranuclear RNA foci (Figure 3F) with an average number of two to five foci per cell for *C9orf72* iPSC-MG and an average number of fewer than one foci per cell for control iPSC-MG (Figure 3G). A notable and significant difference was observed for C9-4 iPSC-MG compared to its isogenic control (Figures 3E–G).

Sense and antisense GGGGCC/CCCCGG repeat-associated non-AUG (RAN) translation produces five different DPR proteins that accumulate in cells and are proposed to contribute to neuronal toxicity and cellular dysfunction (Zu et al., 2013; O'Rourke et al., 2015; Jiang and Cleveland, 2016; Zhang et al., 2016b; Freibaum and Taylor, 2017; Gendron et al., 2017). Here, we assessed the presence of poly-(GP) in mature iPSC-MG cultures by measuring poly-(GP) abundance in cell lysates using a customized immunoassay (Andrade et al., 2020). We detected a significant increase in poly-(GP) levels in *C9orf72* ALS/FTD iPSC-MGs compared to controls, including the isogenic pair C9–4 (Figure 3H), showing for the first time that the *C9orf72* HRE translates into DPR proteins in endogenous *C9orf72* HRE expressing microglial mono-cultures.

Cytoplasmic TDP-43 inclusions are one of the hallmark pathologies of C9orf72 ALS and FTD and have been reported in glial cells of *C9orf72* ALS/FTD postmortem tissues (Al-Sarraj et al., 2011; Cooper-Knock et al., 2012; Schipper et al., 2016). To evaluate whether iPSC-MG mono-cultures exhibit cytoplasmic TDP-43 inclusions, we performed immunocytochemistry for TDP-43 and measured the nucleocytoplasmic (N/C) ratio of TDP-43 using confocal microscopy. No significant difference in the TDP-43 N/C ratio was observed between control and *C9orf72* ALS/FTD microglia at 40 DIV (Supplementary Figures S4A, C). One hypothesis for TDP-43 mislocalization in ALS and FTD is a defect in nucleocytoplasmic trafficking leading to the accumulation of TDP-43 protein in the cytoplasm (Zhang et al., 2015; Chou et al., 2018; Moore et al., 2020). Our laboratory has recently shown that another RNA binding protein, the RNA editing enzyme adenosine deaminase acting on double-stranded RNA 2 (ADAR2), is mislocalized to and accumulates in the cytoplasm of motor neurons in *C9orf72* ALS/FTD (Moore et al., 2019). We, therefore, wondered whether *C9orf72* microglia would display similar ADAR2 mislocalization. Like TDP-43, immunocytochemistry of *C9orf72* ALS/FTD iPSC-MG for ADAR2 revealed no nucleocytoplasmic mislocalization resulting in an unchanged N/C ratio in microglia mono-cultures (Supplementary Figures S4B, D). These data suggest that while *C9orf72* ALS/FTD iPSC-MG mono-cultures do exhibit *C9orf72* pathobiological phenotypes as shown in *C9orf72* ALS/FTD iPSC neurons, there are no microglial cytoplasmic inclusions of TDP-43 or ADAR2.

### IPSC-MG carrying *C9orf72* HRE respond to lipopolysaccharide (LPS) stimulation similarly to healthy control iPSC-MG

**Figure 4:**
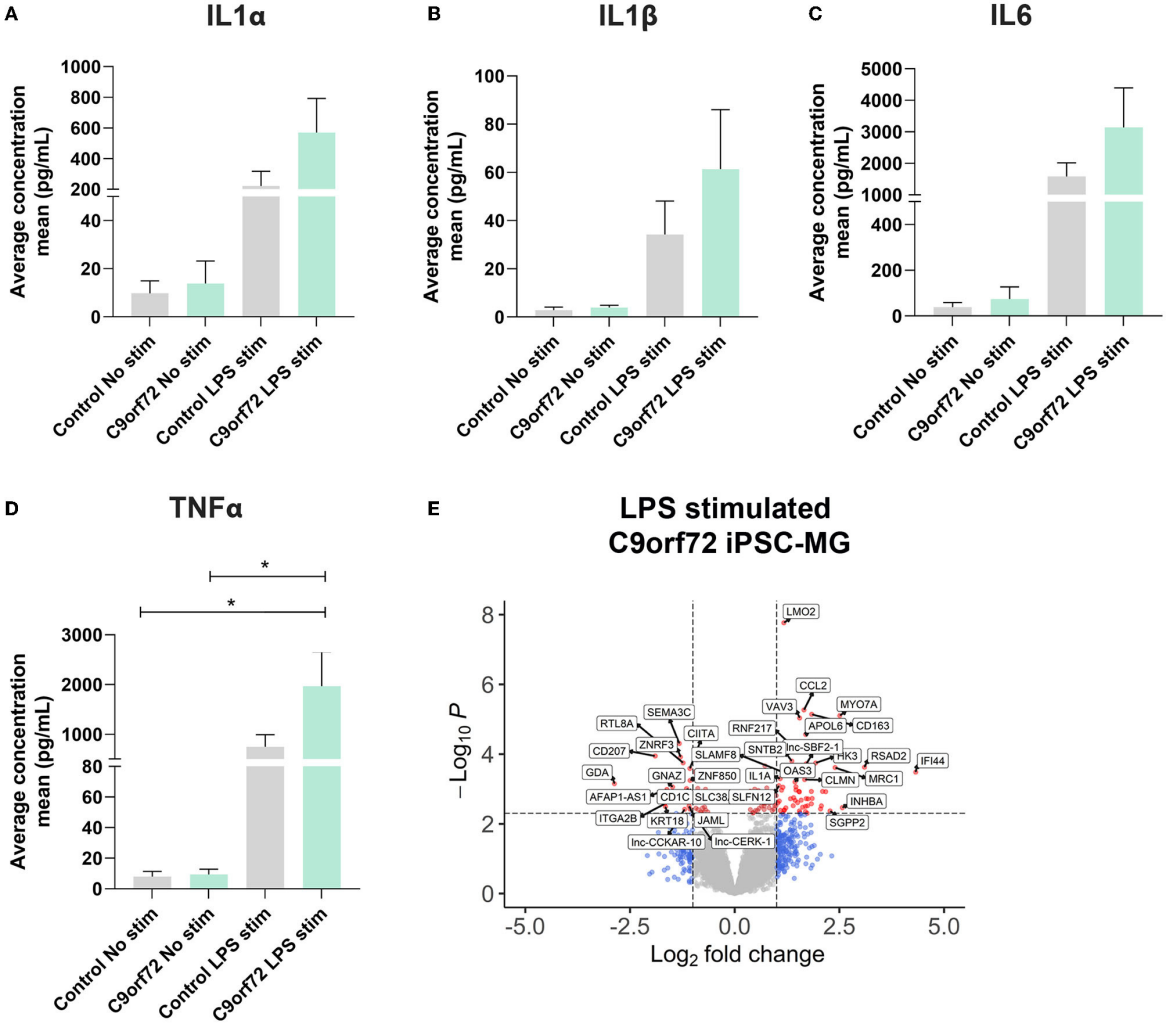
*C9orf72* ALS/FTD and healthy control iPSC-MG show equal response to LPS stimulation. **(A–D)** Cytokine/chemokine profile analysis of iPSC-MG using the U-plex Multiplex Assay. *C9orf72* ALS/FTD iPSC-MG mono-cultures respond with an increase in IL1α, IL6, and TNFα inflammatory cytokines similar to controls (see Supplementary Figures S5A–D for response per replicate within a sample). Data presented as the average concentration of secreted proteins (pg/ml), Mean ± SEM, *n* = 4 control lines, 2–3 replicates; *n* = 3 *C9orf72* lines, three replicates. *P*-values displayed in the graphs; ^*^p ≤ 0.05; two-way ANOVA followed by Tukey's Test and *post hoc* analysis. **(E)** Volcano plot showing differentially expressed genes in *C9orf72* ALS/FTD (*n* = 4) from 6 h LPS stimulation when compared to healthy control (*n* = 5) (Log2 fold change (FC) ± 1, p-value <0.005; see Supplementary Figure S5E).

Microglia can exacerbate or promote neurodegeneration by releasing pro-inflammatory cytokines, including interleukin-1 (IL1), interleukin-6 (IL6), and tumor necrosis factor-α (TNFα) or anti-inflammatory cytokines such as IL4 and IL10 (Smith et al., 2012; Moreno-Martinez et al., 2019; Olesen et al., 2020). As a result, neuroinflammation is considered a major contributor to neuronal dysfunction in neurodegenerative diseases, including ALS/FTD (Lall and Baloh, 2017; Beers and Appel, 2019; McCauley and Baloh, 2019; Olesen et al., 2020). To investigate if *C9orf72* ALS/FTD iPSC-MG respond differently to extracellular stimuli, we treated four controls and three *C9orf72* ALS/FTD lines of iPSC-MGs with the bacterial toxin, LPS, commonly used to evoke a general non-specific immune response *in vitro* and *in vivo* (Beurel and Jope, 2009; Abud et al., 2017; Furube et al., 2018; Hong et al., 2020). Following LPS treatment, we tested for the presence of released chemokines and cytokines in the iPSC-MG cell culture supernatants, as described (Abud et al., 2017). Upon LPS stimulation, both control and *C9orf72* ALS/FTD iPSC-MG mono-cultures exhibited increased release of IL1α, IL1β, IL6, and TNFα compared to basal conditions but no differences between the experimental groups were observed (Figures 4A–D; individual cell response shown in Supplementary Figures S5A–D). These results confirm the ability of *C9orf72* ALS/FTD patient-derived iPSC-MG mono-cultures to respond to extracellular stimuli and support previous *in vitro* studies showing no significant changes in inflammatory responses in murine microglia cell lines overexpressing *C9orf72* HRE (Rostalski et al., 2020). We then treated five controls and four *C9orf72* ALS/FTD lines of iPSC-MG with LPS and analyzed gene expression alterations by RNA sequencing. Both, healthy control and *C9orf72* ALS/FTD microglia within their experimental group responded to LPS treatment with significant gene expression changes (Supplementary Figures S6A–B; log_2_ fold change (FC) ± 1, *p* < 0.005). In both experimental groups, differential expression was found in genes involved in inflammatory processes and interferon-gamma signaling pathways, e.g., *CXCL8, CXCL10, TNIP3, CCL4L2, CCL20, IFIT2, IFIT3, IL23A, IL12B*, and *IL6*. Among the top LPS-induced significantly dysregulated disease-specific genes is the cytokine IL1α, which is highly expressed and secreted by microglia and is known to be a strong inducer of an A1 reactive astrocyte phenotype (Figure 4E, Supplementary Figure S5E; log_2_ fold change (FC) ± 1, *p* < 0.005) (Zhang et al., 2014; Bennett et al., 2016; Liddelow et al., 2017).

### *C9orf72* ALS/FTD iPSC-MGs exhibit subtle increases in phagocytic uptake of toxic Aβ peptides and present altered transcriptomic profiles after Aβ exposure

**Figure 5:**
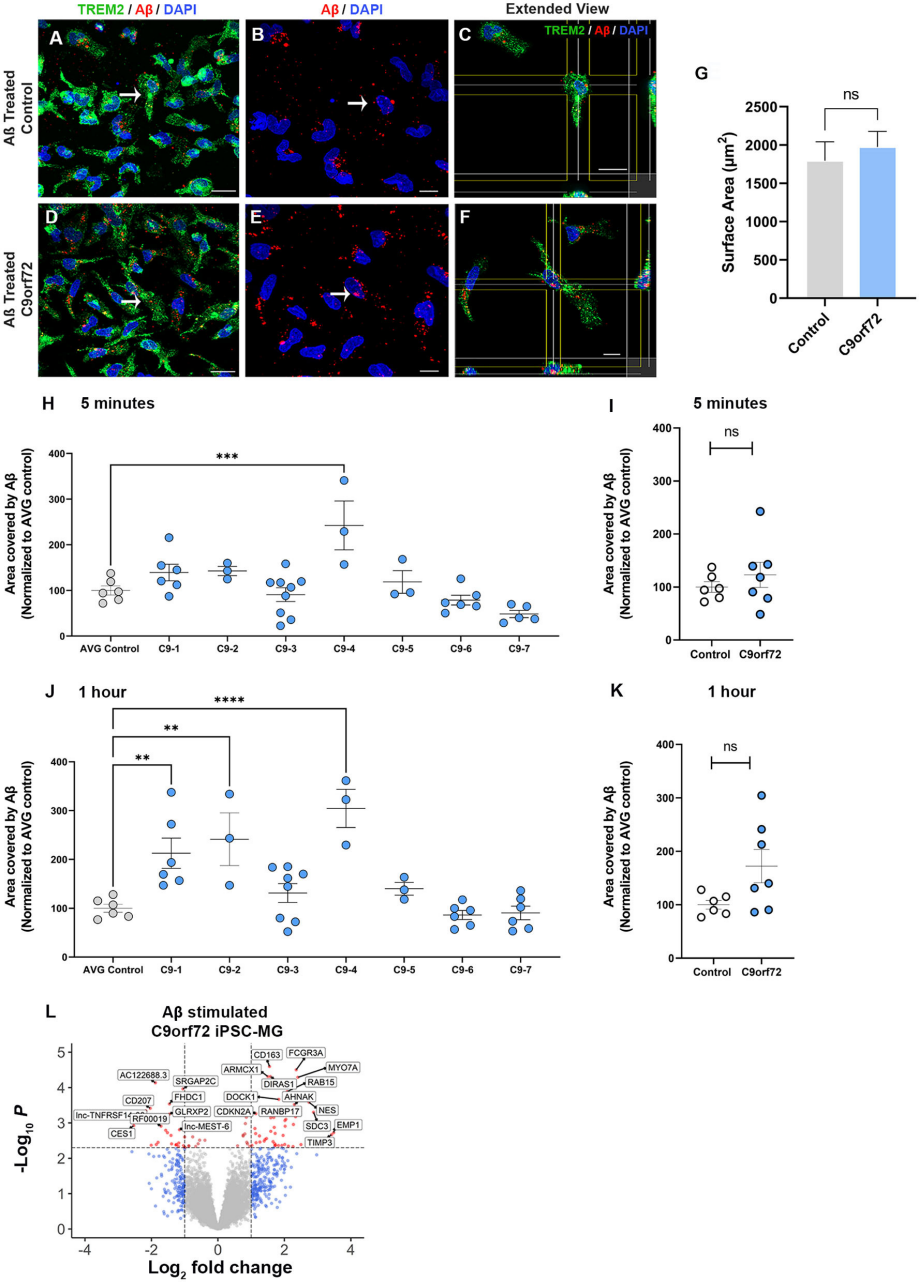
*C9orf72* ALS/FTD iPSC-MGs show increased phagocytosis of synthetic Aβ (1–40) in select patient lines. **(A, D)** Representative images of healthy controls and *C9orf72* ALS/FTD iPSC-MG stained for TREM2 (green) and highlighting Aβ (1–40) TAMRA (red) inside the cells at 30 min. White arrow point at phagocytic cells containing Aβ (1–40) TAMRA (red). Scale bar, 20 μm. **(B, E)** Aβ (1–40) TAMRA (red) internalized by healthy controls and *C9orf72* ALS/FTD iPSC-MG. Scale bar, 20 μm. **(C, F)** Extended view showing phagocytic activity in both iPSC-MG groups. Scale bar, 20 μm. **(G)** No significant differences in the microglia cell surface area were observed upon Aβ exposure (control, *n* = 5 lines; *C9orf72, n* = 6 lines; *n* = 1–2 differentiations per line; 2–3 replicates; 6–7 pictures per replicate; *n* = 6–10 cells per picture; *p* = 0.58 using Student's *t*-test). **(H–K)** Select patient lines showed significant differences in the percentage of cell surface area covered by Aβ (1–40)-TAMRA after 5 min (1 out of 7 lines) or 1 h (3 out of 7 lines) (control, *n* = 6 lines; *C9orf72, n* = 7 lines; *n* = 1–2 differentiations per line; 2–3 replicates; 6–7 images per replicate; *n* = 6–10 cells per image). One-way ANOVA followed by a Dunnett's *post hoc* correction was performed, **p ≤ 0.01; ***p ≤ 0.001; ****p ≤ 0.0001. Note: when grouped, no significance was observed at either time point; therefore, the Student's *t*-test was performed. **(L)** Differentially expressed genes from *C9orf72* ALS/FTD Aβ (1–40) treated iPSC-MG (control, *n* = 5 lines; *C9orf72, n* = 4 lines; Log2 fold change (FC) ± 1, *p* < 0.005; see Supplementary Figure S6E).

A major function of microglia is to phagocytose unwanted toxic substances and cell debris that can negatively impact brain function. To determine whether control and *C9orf72* ALS/FTD iPSC-MG differ in their phagocytic activity and their ability to degrade toxic products, we transiently treated iPSC-MGs with a fluorescently tagged synthetic amyloid beta (Aβ) cargo, known to be phagocytosed by microglia (Paresce et al., 1996; Parnaik et al., 2000; Paolicelli et al., 2017). We treated five healthy control and seven C9orf72 ALS/FTD lines of iPSC-MGs for 5 min with 1 μM Aβ (1-40)-TAMRA or vehicle and then assessed engulfment and clearance at 5 min and after 1 h. To quantify phagocytic activity, iPSC-MG were fixed and immunostained for TREM2, a known immune receptor selectively expressed in microglia (Figures 5A–F). After confocal microscopy and image analyses, we calculated the percentage of iPSC-MG surface area covered by Aβ (1-40)-TAMRA protein in individual cells at both time points. We observed varying levels of retention of phagocytosed Aβ depending on the patient lines (Figures 5H, J). At the 5 min time point, only one *C9orf72* line retained significantly more Aβ (1-40)-TAMRA when compared to healthy controls. At the 1 h time point, three out of seven *C9orf72* patient lines showed significantly increased area covered by Aβ when compared to healthy control lines. Increased retention of intracellular Aβ levels could suggest potential dysfunction of the endosomal–lysosomal pathways and an inability to adequately process phagocytosed cargo in select patient lines (Figures 5H, J). When grouped, no differences in phagocytosed content between four *C9orf72* ALS/FTD and healthy control iPSC-MG were noted (Figures 5I, K). To take into account possible changes in cell size, we measured the total microglia cell surface. No significant differences in the surface area between experimental groups were observed (Figure 5G). Finally, we wondered whether microglia exposed to Aβ would alter their transcriptional profile. Therefore, a separate set of five control and four *C9orf72* ALS/FTD lines of iPSC-MGs were treated with Aβ (1-40)-TAMRA and analyzed for gene expression changes via RNA sequencing analyses. Despite lowering the stringency of our DEG analyses to a *p* < 0.05, neither control nor disease iPSC-MG showed large numbers of dysregulated genes when we compared treated to untreated cells within each experimental group (Figures S6C, D; log_2_ fold change (FC) ± 1, *p* < 0.05). However, when we examined DEG in Aβ-treated *C9orf72* ALS/FTD iPSC-MG in reference to Aβ-treated control microglia, we noted several significantly differentially expressed genes [69 genes; log_2_ fold change (FC) ± 1, *p* < 0.005; Figure 5L, Supplementary Figure S6E]. Among the upregulated genes were *FCGR3A* and *DOCK*, which are genes known to play a role in phagocytosis (Wu and Horvitz, 1998; Sivagnanam et al., 2010). Additional upregulated genes, such as *RAB15, AHNAK*, and *SDC3*, play a role in endocytic trafficking, cytoskeletal rearrangement, or cell migration (Carey, 1996; Zuk and Elferink, 2000; Dumitru et al., 2013). These results suggest that *C9orf72* ALS/FTD iPSC-MG responds to Aβ stimuli with an altered transcriptional response of select gene pathways.

### *C9orf72* ALS/FTD iPSC-MGs engulf human healthy brain synaptoneurosomes similar to control iPSC-MGs

**Figure 6:**
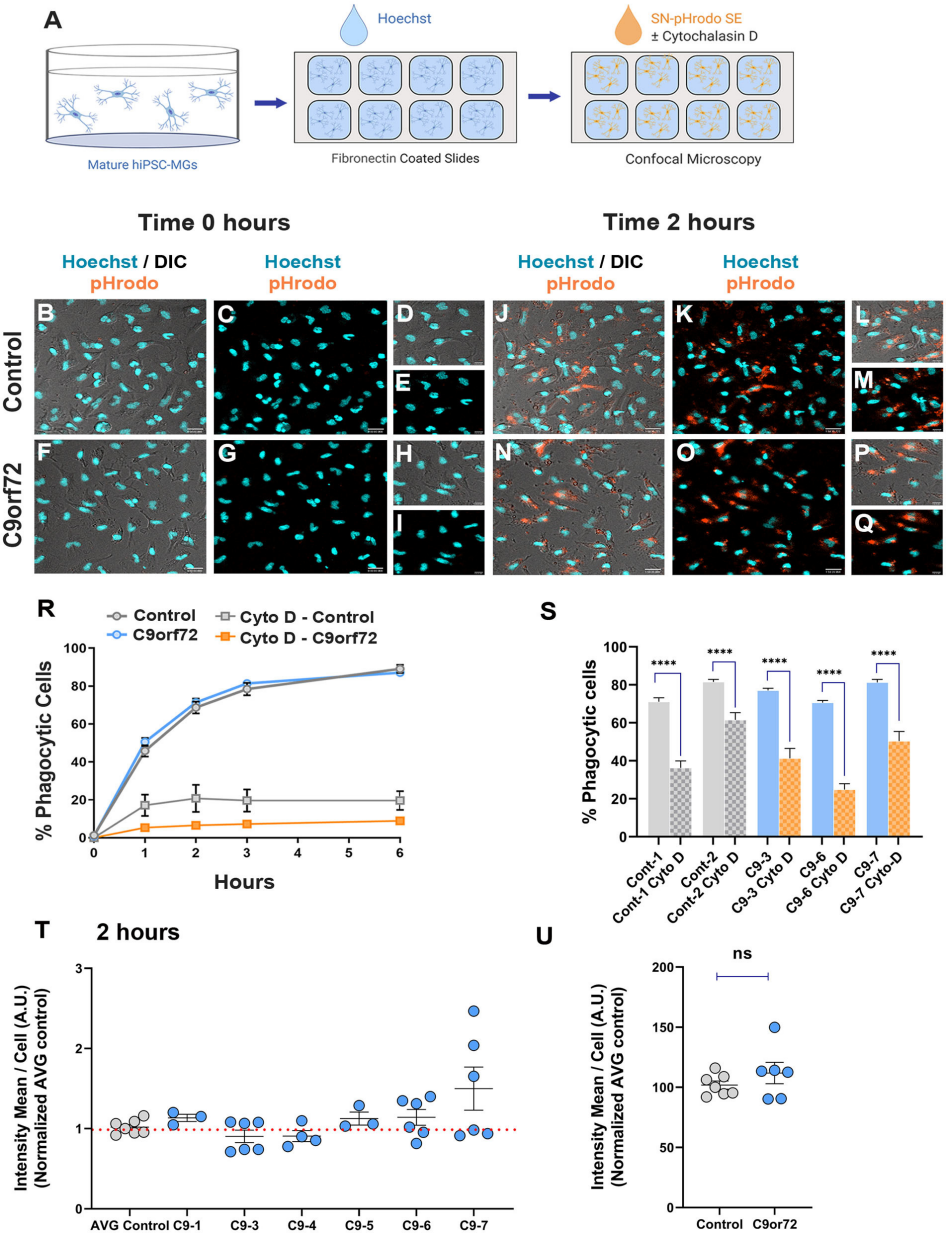
Phagocytic uptake of human brain synaptoneurosomes by iPSC-MGs. **(A)** Illustration of iPSC-MGs treated with human brain synaptoneurosomes. Cultured control and *C9orf72* ALS/FTD iPSC-MGs (Day 40) are plated onto fibronectin-coated slides and labeled with Hoechst nuclear marker followed by hSN-rodo treatment ± 10 μM Cytochalasin D (actin polymerization inhibitor). **(B–I)** Control and *C9orf72* ALS/FTD iPSC-MG images were taken at 0 h time point. IPSC-MGs labeled with live nuclear stain Hoechst (cyan) and exposed to hSN-rodo (orange). Differential interference images (DIC) enhance the visualization of individual iPSC-MG morphology **(B, F)**. No evident phagocytosis of hSN-rodo is seen at this time point **(C, G)**. Scale bars = 50 μm. Higher magnification representative images at 0 h time point of control **(D, E)** and *C9orf72* ALS/FTD iPSC-MGs **(H, I)**. Scale bars = 10 μm. **(J–Q)** Representative images of control and *C9orf72* ALS/FTD iPSC-MG at 2 h show an increase in hSN-rodo fluorescent signal inside iPSC-MG indicative of phagocytosis and uptake into intracellular acidic compartments. Scale bar = 40 μm. **(L, M, P, Q)** Higher magnification representative images highlighting the increase in hSN-rodo signal in individual iPSC-MG at 2 h. Scale bar = 10 μm. **(R)** Percentage of iPSC-MG engulfing hSN-rodo during the 6 h initial time course of live imaging. At 2 h, control and *C9orf72* iPSC-MG showed 69% and 71% phagocytic activity, respectively. No significant differences in synaptoneurosome uptake were observed between groups. Approximately 10 uM cytochalasin D, an actin polymerization inhibitor, was used as a negative control to inhibit phagocytic activity in iPSC-MGs (For 6 h initial time course; control, *n* = 1 line; *C9orf72, n* = 2 lines; 3 replicates; *n* = 6 images/replicates/time points; *n* = 8 cells per image for hSN-rodo; for cytochalasin D, *n* = 6 images/groups/time points; *n* = 10 cells per image). **(S)** Percentage of iPSC-MG engulfing hSN-rodo at 2 h time point with cytochalasin D treatment. A significant decrease in phagocytic activity was observed in controls and *C9orf72* ALS/FTD iPSC-MG in the presence of cytochalasin D (Cyto-D, control, *n* = 2 lines; *C9orf72, n* = 3 lines, n =6 images/group; ****p ≤ 0.0001 using Student's *t*-test). **(T, U)** HSN-rodo mean intensity per cell normalized to average control at 2 h (control, *n* = 7 lines; *C9orf72, n* = 6 lines; 3 replicates; 6–7 images per replicate; *n* = 6–10 cells per image). Each iPSC-MG line was normalized and compared to the average controls. One-way ANOVA statistical analysis showed no significant differences in phagocytosis of control human brain synaptoneurosomes between groups. Student's *t*-test showed no significant differences when grouped. Data presented as Mean intensity ± SEM.

An important aspect of microglia–neuron communication in neurodegeneration is the role of microglia in the maintenance and refinement of synaptic networks through the selective pruning of synapses. This process occurs predominantly during development (Stevens et al., 2007; Tremblay et al., 2011; Bialas and Stevens, 2013). However, synaptic pruning pathways are known to be re-activated in neurodegeneration leading to synapse loss and contributing to cognitive impairments (Henstridge et al., 2016; Hong et al., 2016; Lui et al., 2016; Colom-Cadena et al., 2020). To determine if *C9orf72* ALS/FTD iPSC-MG can phagocytose synapses or exhibit altered phagocytosis due to the presence of *C9orf72* HRE, we exposed iPSC-MG mono-cultures to synaptoneurosomes (hSN) derived from healthy control human brain and assessed synaptoneurosomes engulfment via live confocal microscopy. Fresh frozen control human postmortem tissues were used to prepare hSN (Hesse et al., 2019; Tzioras et al., 2019). Synaptic fractions were enriched for pre-synaptic protein synaptophysin and the post-synaptic density 95 (PSD-95) compared to total brain homogenate and nuclear marker histone 3 (Supplementary Figures S7A, B). Control and *C9orf72* ALS/FTD iPSC-MGs were labeled with the live cell nuclear marker Hoechst (Figures 6A–I) to identify individual cells followed by treatment with hSN fluorescently tagged with pHrodo succinimidyl ester (hSN-rodo; Figures 6A, J–Q). An increase in pHrodo fluorescence is indicative of the uptake of hSN-rodo into acidic intracellular compartments of iPSC-MGs. After an initial 6-h live cell imaging to determine an optimal time point of internalization of hSN-rodo in control and *C9orf72* ALS/FTD iPSC-MGs, we observed that more than 60% of cells exhibit phagocytic activity at 2 h (Figure 6R). IPSC-MG phagocytosis was reduced significantly for both control and *C9orf72* ALS/FTD groups in the presence of cytochalasin-D, an actin polymerization inhibitor known to inhibit phagocytosis (Figures 6R, S). We then treated iPSC-MGs (control, *n* = 7 lines; C9orf72, *n* = 6 lines; three replicates; six to seven images per replicate) with hSN-rodo and performed fluorescent live cell imaging for 2 h, capturing images every 10 min (Supplementary Figure S7C, Supplementary Video S1). The mean intensity of hSN-rodo was similarly increased in *C9orf72* ALS/FTD iPSC-MGs compared to controls (Figures 6T, U). Together, these data suggest that *C9orf72* ALS/FTD iPSC-MGs exhibit typical microglia phagocytic activity as shown by the engulfment of healthy human brain synaptoneurosomes. Additional studies are required to determine if *C9orf72* ALS/FTD iPSC-MGs respond differently to diseased human brain synaptoneurosomes, which may contain specific signaling molecules necessary for microglia activation and elimination of synapses.

## Discussion

An extensive body of evidence has suggested that glia contributes to the neurodegeneration observed in ALS and FTD (Yamanaka et al., 2008; Ilieva et al., 2009; Ban et al., 2019; Valori et al., 2019; Dols-Icardo et al., 2020; Filipi et al., 2020). Transcriptional assessments and proteomic approaches across the ALS/FTD spectrum using predominantly postmortem autopsy tissue have reported robust glia signatures and glia protein modules, respectively, emphasizing glial cell involvement in inflammation and contribution to disease (D'Erchia et al., 2017; Umoh et al., 2018; Tam et al., 2019; Dols-Icardo et al., 2020).

In the present study, we generated microglia mono-cultures from mutant *C9orf72* ALS/FTD patient-derived iPSC to evaluate their cellular and molecular phenotypes and determine any functional and/or pathological characteristics that might contribute to neurodegeneration in *C9orf72* ALS/FTD. The differentiation protocol was selected based on the transcriptional and functional similarities between the generated human iPSC-MG to adult human microglia, as well as for their high purity, yield, and distinction from other myeloid cells, such as monocytes and dendritic cells (Abud et al., 2017; McQuade et al., 2018). The *C9orf72* ALS/FTD iPSC-MG displayed typical microglia characteristics and presented a unique transcriptomic signature profile compared to iPSC-CNs or other glial cell types (Zhang et al., 2014). Applying a list of human microglia-enriched genes from Gosselin and colleagues (Gosselin et al., 2017), transcriptional analyses revealed no significant differences between these microglial-enriched genes between *C9orf72* ALS/FTD and control iPSC-MG under basal, unstimulated culture conditions. This supports the notion that the presence of the *C9orf72* HRE does not affect iPSC microglia differentiation and their cell-type specific transcriptome. We did detect overall, non-microglia-enriched differentially expressed genes in the *C9orf72* iPSC-MG (Figure 2), including the downregulation of formyl peptide receptor 3, *Fpr3*. While little is known about the role of *Fpr3* in microglial function and activation, its paralog *Fpr2* has been implicated in inflammatory responses to Aβ plaques in AD (Le et al., 2001; Tiffany et al., 2001; Cui et al., 2002; Ries et al., 2016). Interestingly, a recent study in murine BV2 microglia cell lines showed that activation of FPR2 reversed Aβ-induced microglial activation and subsequent apoptosis of SH-SY5Y-differentiated neurons (Wickstead et al., 2020).

As microglial function is influenced by the cellular environment, we examined changes in the previously reported microglial-enriched genes (Gosselin et al., 2017) using existing bulk RNA sequencing data from postmortem *C9orf72* ALS/FTD brain tissues. Surprisingly, minimal changes in the microglial-enriched genes were found either in the frontal, motor, or occipital cortex supporting the need for single-cell resolution technologies to better identify changes in gene expression in specific cell populations, similar to what has been reported in Alzheimer's disease brain tissues' analyses (Keren-Shaul et al., 2017; Mrdjen et al., 2018; Bottcher et al., 2019; Masuda et al., 2019, 2020; Sankowski et al., 2019). Cell type-specific analyses from postmortem *C9orf72* ALS/FTD brain tissue will further allow for the identification of subsets of microglial subpopulations and associate their transcriptional signatures with potential neuroprotective or detrimental roles, as well as microglial-specific disease pathways and mechanisms.

To our knowledge, these data are the first to indicate that human endogenous *C9orf72* ALS/FTD iPSC-MG exhibits intrinsic *C9orf72* pathology. Although, transcriptional analyses indicated variability of *C9orf72* mRNA levels across *C9orf72* ALS/FTD iPSC-MG patient lines, no significant differences in *C9orf72* expression were observed by RNA sequencing or quantitative RT-PCR analysis. Our data are consistent with previous studies in iPSC patient-derived neurons and astrocytes (Sareen et al., 2013; Zhao et al., 2020). A larger sample size might be required for RNA sequencing analysis to obtain significant results. As for the qRT-PCR, due to the existence of several *C9orf72* RNA variants and the notion that there is cell type-specific promoter usage of *C9orf72* variants, the primers used for the present qRT-PCR might not reflect a microglia-specific reduction in *C9orf72* gene expression (Sareen et al., 2013). We observed a significant decrease in C9orf72 protein expression in *C9orf72* iPSC-MG, which is an interesting finding, as previous reports on *C9orf72* iPSC-astrocytes showed no reduction in C9orf72 protein levels (Zhao et al., 2020). The loss of function of C9orf72 protein has been implicated in alterations of endosomal–lysosomal pathways, hence could contribute to the phagocytic differences we observed among individual *C9orf72* patient lines (Farg et al., 2014; Amick et al., 2016, 2020; O'Rourke et al., 2016; Sullivan et al., 2016; Shi et al., 2018).

We also evaluated *C9orf72* iPSC-MG for the non-canonical translation of DPR proteins, specifically poly-(GP), and are the first to report endogenous poly-(GP) production in patient *C9orf72* microglia, suggesting that similar to *C9orf72* iPSC-astrocytes, microglia undergo repeat-associated non-ATG translation (Zhao et al., 2020). Recent studies have shown neuron-astroglia transmission of *C9orf72*-associated DPRs via exosomes in an *in vitro* culture system (Westergard et al., 2016). It is yet to be determined whether other DPRs are present in *C9orf72* iPSC-MG and whether, similarly to astrocytes, microglia contribute to the transmission of DPRs to neighboring cells. Most recent studies showed that overexpression of Proline–Arginine DPR protein in the HMC3 human microglia cell model is associated with significant induction of NLRP3 inflammasome activity (Fu et al., 2022). The authors further showed that conditioned media from this human microglia cell line caused neuronal cell damage, emphasizing a significant role of microglia activity in neurodegeneration.

*C9orf72* DPRs have been suggested to contribute to nucleocytoplasmic trafficking defects present in *C9orf72* ALS/FTD neurons (Jovicic et al., 2015; Boeynaems et al., 2016; Moore et al., 2020). One of the consequences of these trafficking defects is the mislocalization of nuclear RNA-binding proteins, such as TDP-43. TDP-43 pathology is present in the glia of *C9orf72* postmortem tissues (Cooper-Knock et al., 2012; Brettschneider et al., 2014; Fatima et al., 2015; Schipper et al., 2016; Yamanaka and Komine, 2018). TDP-43 has further been associated with neuroinflammation, microglia neuroprotection, and the regulation of microglia phagocytosis (Swarup et al., 2011; Paolicelli et al., 2017; Spiller et al., 2018). In the present study, TDP-43 cytoplasmic accumulations or loss of nuclear TDP-43 was not detected in *C9orf72* ALS/FTD iPSC-MG mono-cultures; similar to what has been observed in *C9orf72* iPSC patient-derived astrocytes (Zhao et al., 2020). Furthermore, we found no significant difference in the nucleocytoplasmic ratio of the RNA editing protein ADAR2, which has recently been shown to be mislocalized to the cytoplasm of *C9orf72* ALS/FTD iPSC motor neurons, as well as in neurons in *C9orf72* ALS/FTD postmortem tissues and *C9orf72* ALS/FTD mouse model brain tissues (Moore et al., 2019). The absence of TDP-43 and ADAR2 mislocalization could be due to the cellular age of the differentiated cells, as recent human postmortem tissue studies suggested that TDP-43 mislocalization is a late-stage event of C9orf72 ALS pathogenesis (Vatsavayai et al., 2016).

Cerebral spinal fluid and blood cytokine profiles are significantly altered for a large array of cytokines and chemokines in ALS/FTD (Lu et al., 2016). In addition, recent data support a correlation between specific immune responses of gene-associated ALS subgroups and patient survival (Olesen et al., 2020). As for *C9orf72* ALS/FTD, previous studies in *C9orf72-*deficient mice revealed increased IL6 and IL1β mRNA levels in microglia and upregulation of inflammatory pathways, suggesting an association among the loss of *C9orf72*, altered microglia function, and pro-inflammatory phenotypes (Lagier-Tourenne et al., 2013; Prudencio et al., 2015; O'Rourke et al., 2016; Lall et al., 2021). Here, *C9orf72* ALS/FTD iPSC-MG mono-cultures exhibit a comparable response to control iPSC-MG upon LPS stimulation. Further studies are necessary to determine if *C9orf72* ALS/FTD iPSC-MG present or exacerbate an inflammatory phenotype when stimulated to induce more specific inflammatory responses, such as activation of the inflammasome via the NLRP pathway (Fu et al., 2022; Trageser et al., 2023) or co-stimulation of LPS and IFNγ to specifically activate pro-inflammatory pathways (Kann et al., 2022). Furthermore, it will be interesting to examine inflammatory responses when iPSC-MG are co-cultured with diseased neurons or astrocytes knowing that direct contact of iPSC-MG with CNS cells can influence their gene expression (Abud et al., 2017). Similarly, it will be important to determine if an anti-inflammatory response is acquired in the presence of *C9orf72* ALS/FTD iPSC-neurons or other glial cell types.

Overall, we report intrinsic properties of *C9orf72* ALS/FTD iPSC-MG mono-cultures and set the stage for the use of this human endogenous disease model in co-cultures with *C9orf72* ALS/FTD iPSC derived neurons and other glial cells, astrocytes, and/or oligodendrocytes. *C9orf72* ALS/FTD iPSC-MG can be used in a 2- or 3-dimensional co-culture system or can be transplanted *in vivo* into mouse models to further assess the microglial contribution to neuronal dysfunction and degeneration in *C9orf72* ALS/FTD (Abud et al., 2017; Xu et al., 2020). Finally, this human cell culture model provides novel opportunities to find mechanisms of disease and to screen microglial-targeted new therapeutics for future drug development for ALS/FTD patients.

## Materials and methods

### IPSC lines

The majority of the iPSC lines used in our studies were purchased from Cedars Sinai-induced pluripotent stem cell core https://www.cedars-sinai.edu/research/areas/biomanufacturing/ipsc.html or obtained from collaborators who have characterized the lines in previous publications. All purchased iPSC lines from Cedars Sinai are characterized for (1) positive staining of pluripotency markers (Oct3/4, NANOG, SOX2, TRA-1-60, TRA-1-81, and SSEA4); (2) karyotyping to reveal no chromosomal abnormalities; (3) determine a pluripotency score via PrimeView global gene expression profile assay (PluriTest); (4) trilineage differentiation potential via Taqman hPSC scorecard assay to confirm appropriate expression of ectodermal, endodermal, and mesodermal factors in the iPSC cells; (5) cell line authentication analysis—STR Analysis—to confirm identity/profile matching score with primary tissue. A Certificate of Analysis (COA) for these lines is available upon request. Please see Supplementary Table S1 for comprehensive information on the lines used in these studies.

### Generation of hematopoietic progenitor cells (HPCs) DIV −1 to DIV 12

IPSCs were differentiated into microglia following an established protocol (McQuade et al., 2018). In brief, iPSCs were maintained in mTeSR Plus Kit (Stemcell Technologies # 05825) in 10 cm dishes. At DIV−1 of HPCs differentiation, 5- to 7-day-old iPSC cultures were then used to generate a cluster of differentiating 43 positive (CD43^+^) hematopoietic progenitor cells (HPCs) following a 12-day commercially available kit (STEMdiff Hematopoietic Kit; Stemcell Technologies # 05310). IPSCs were cleaned and 1/3 dish was gently dissociated with dispase (Stemcell Technologies # 07923) for 12–15 min at 37°C. IPSCs were then collected and spun down at 500 rpm for 1–2 min and resuspended in 2 mL of mTeSR Plus media with 20 μM ROCK inhibitor Y-27632 (Stemcell Technologies # 72304). Matrigel, hESC-Qualified Matrix (Corning # 354277), coated six-well plates were used to start the HPC differentiation. Using a 5-mL serological pipette, one drop or two drops of iPSCs were seeded per well into matrigel. Then, the next day, on DIV 0 of HPCs differentiation, wells with 80 small colonies per well were selected to start HPCs differentiation. IPSCs were fed following the manufacturer's instructions. On DIV 12 of HPCs differentiation, only the non-adherent HPCs were transferred to a new six-well plate to start microglia differentiation (DIV 12/0).

### Differentiation of HPCs into microglia cells DIV 12/0—DIV 40/28

Matrigel, GFR (growth factor reduced) Membrane Matrix (Corning # 356231), coated six-well plates were prepared to start microglia differentiation (DIV12/0). HPCs were differentiated into microglia for 28 days using serum-free media conditions. HPCs in suspension were collected and spun down at 300 xG for 6 min and resuspended into microglia basal media (MBM, 2 mL/well) containing: DMEM/F12 no phenol (Gibco # 11-039-021), 2% Insulin Transferin Selenite (Gibco # 41400045), 2% B27 (Gibco # 17504-044), 0.5% N2 (Gibco # 17502-048), 1% Glutamax (Gibco # 35050-061), 1% NEAA (Gibco # 11140-050), 1% Pen/Strep (Gibco # 15140-122), 400 μM 1-Thioglycerol (Sigma # M1753), 5 μg/mL human insulin (Sigma # I2643), and supplemented with three growth factors (GFs): 100 ng/mL human recombinant interleukin-34 (IL-34; Peprotech # 200-34), 25 ng/mL macrophage colony-stimulating factor (M-CSF; Gibco # PHC9501), and 50 ng/mL transforming growth factor β1 (TGFβ1; Miltenyi Biotec # 130-108-969) (MBM + 3GFs); cytokines and growth factors are known to be essential for the development of microglia (Chen et al., 2002; Abutbul et al., 2012; Greter et al., 2012; Wang et al., 2012; Butovsky et al., 2014; Elmore et al., 2014; Abud et al., 2017; Pandya et al., 2017). Microglia differentiation starts (DIV 12/0) once the HPCs are transferred and plated at a density of 200 000 cells per well of a six-well plate. Cells will predominantly grow in suspension. On DIV 2, 4, 6, 8, and 10 of microglia differentiation, 1 mL of MBM + 3GFs media was added to each well. On DIV 12, a partial media change was done. IPSC-MGs from one six-well plate were spun down at 300 xG for 6 min and resuspended into MBM + 3 GFs and split back into the same six-well plate. On DIV 14, 16, 18, 20, 22, and 24, 1 mL of media was added per well. On DIV 25, MBM + 3GFs was changed to maturation media composed of MBM supplemented with the five growth factors (MBM +5 GFs): 100 ng/mL IL34, 25 ng/mL M-CSF, 50 ng/ml TGFβ1, 100 ng/mL cluster of differentiation 200 (CD200; Novoprotein # C31150UG), and 100 ng/mL fractalkine chemokine C-X3-C motif ligand 1 (CX3CL1; Peprotech # 300-31) (MBM + 5 GFs). The presence of CD200 and CX3CL1 in the culture media, both glial and neuronal molecules, is critical for microglia maturation and maintenance of an *in vivo-*like microglia resting state phenotype in an *in vitro* setting (Barclay et al., 2002; Kim et al., 2011; Kierdorf and Prinz, 2013) (Figure 1A). At DIV 28, IPSC-MG reached maturation. Moreover, 1 mL of MBM + 5GFs media was added to the cultures every other day. Mature cells were used for experimentation within 10 days (DIV 28–DIV38).

### Differentiation of iPSCs into cortical neurons

For cortical neuron differentiation, 70% of confluent iPS cells maintained in mTeSR Plus media on 10 cm dishes were used for embryoid bodies (EBs) formation. The cells were cultured in low attachment six-well plates (Greiner bio-one # 657970) using WiCell Medium containing: DMEM/F12 (Gibco #11330057), 25% knock-out serum replacement (Gibco # 10828-028), 1.3% L-glutamine (Gibco # 35050-061), 1.3% NEAA (Gibco # 11140-050), and 0.1 mM 2-Mercaptoethanol (Sigma# M3148), and placed on a shaker in the incubator for 8 days to allow EBs formation. EBs were then resuspended in Forebrain Neural Induction Media (FB-NIM) containing: DMEM/F12, 1% N2 supplement (Gibco # 17502-048), 1% NEAA, 2 ug/mL heparin (Sigma # H3149), and 10 μg/mL bFGF (Stemcell Technologies # 78003), and plated on to T25 flasks coated with basement membrane matrigel (Corning # 356234) to allow formation of neuronal rosettes. Neuronal rosettes were maintained in FB-NIM for the next 10 days and then, collected and maintained in suspension on a shaker with half FB-NIM media changes every other day to allow for neurosphere growth. Neurospheres were maintained for 20 days in FB-NIM and then resuspended using forebrain neuronal differentiation media (FB-DM) containing: Neurobasal Medium (Corning # 21103-049), 2% B27 (Gibco # 17504-044), 10 ug/mL BDNF (Stem cell technologies # 78005), 10 ug/mL GDNF (Stem cell technologies # 78058), 1 μg/mL laminin (Life technologies # 23017-015), 3.3 μg/mL cAMP (Stem cell technologies # 73884), 3.52 μg/mL Ascorbic acid (Stem cell technologies # 72132), 0.5 mM L-glutamine, and 1% NEAA, and then plated on to T25 flasks. iPSC cortical neurons were harvested on DIV 65-72 for RNA sequencing analysis.

### Immunocytochemistry of iPSC-MG

For iPSC-MG DIV 28–33 of microglia differentiation, non-adherent iPSC-MGs were collected and plated onto a 4- or 8-well fibronectin (sigma # F0895; 1:40) coated glass bottom chamber slides (Ibidi # 80427 or #80827) at a seeding density of 250 000 or 125 000 cells per well, respectively. One hour after plating, cells were fixed with 4% paraformaldehyde (PFA; Electron Microscopy Sciences # 15714-S) for 20 min, washed three times in PBS for 5 min, and then blocked with 0.2% Triton X-100 and 5% Normal Goat Serum (Vector # S1000) for 1 h at room temperature. Primary antibodies were prepared in a blocking solution and applied overnight at 4°C. The following primary antibodies were used during our studies: anti-PU.1 (Cell Signaling Technology # 2266S) 1:500; anti-P2RY12 (Sigma # HPA014518) 1:500; anti-CX3CR1 (Biorad/AbD Serotec # AHP1589) 1:500; anti-TREM2 (Abcam # AB209814) 1:500; anti-TMEM119 (Abcam # ab185333) 1:100; anti-LAMP1 (Developmental Hybridoma Bank # H4A3-s) 1:100; anti-EAA1 (BD Biosciences # 610457) 1:700; anti-C9orf72 (Sigma # HPA023873) 1:100; anti-TDP-43 (Cell signaling # 89789, TDP-43 D9R3L) 1:500; and anti-ADAR-2 (Sigma # HPA018277) 1:500. Next, cells were washed in PBS three times for 7 min and then incubated consecutively with respective fluorophores secondary antibodies. Alexa Fluorophores (Invitrogen) were used at a 1:750 and prepared in blocking solution without triton and incubated for 45 min at room temperature. Cells were then washed with PBS three times for 7 min each and DAPI was applied. For nuclear markers TDP-43 and ADAR, wheat germ agglutinin 680 (Invitrogen # W32465) was used to label the iPSC-MG cell surface. Mounting media ibidi (ibidi # 50001) was used on chamber slides.

### RNA isolation, whole transcriptome library preparation, and sequencing

At DIV 28–30, iPSC-MG from eight *C9orf72* ALS/FTD patient lines and four control lines were pelleted and lysed using QIAshredder (QIAGEN-79654) and RNA was isolated with RNeasy Mini Kit (QIAGEN-74104) following the manufacturer's instructions. RNA samples were measured for quantity with Quant-iT Ribogreen RNA Assay (Thermo Fisher, Cat. No. R11490) and quality with Agilent High Sensitivity RNA ScreenTape and buffer (Agilent, Cat. No. 5067-5579 & 5067-5580). For each RNA sample, an indexed, Illumina-compatible, double-stranded cDNA whole transcriptome library was synthesized from 1 μg of total RNA with Takara Bio's SMARTer Stranded Total RNA Sample Prep Kit – HI Mammalian (Takara Bio, Cat. No. 634876) and SMARTer RNA Unique Dual Index Kit (Takara Bio, Cat. No. 634418). Library preparation included ribosomal RNA depletion, RNA fragmentation (94°C for 3 min), cDNA synthesis, and a 12-cycle unique dual indexing enrichment PCR. Each library was measured for size with Agilent's High Sensitivity D1000 ScreenTape and reagents (Agilent, Cat. No. 5067–5584 and 5067–5603) and concentration with KAPA SYBR FAST Universal qPCR Kit (Kapa Biosystems, Cat. No. KK4824). Libraries were then combined into an equimolar pool which was also measured for size and concentration. The pool was clustered onto a paired-end flowcell (Illumina, Cat. No. 20012861) with a 20% v/v PhiX Control v3 spike-in (Illumina, Cat. No. FC-110-3001) and sequenced on Illumina's NovaSeq 6000. The first and second reads were each 100 bases. All Aβ (1–40) and LPS-stimulated cells at DIV 28-30 iPSC-MG from four *C9orf72* ALS/FTD patient lines and five control lines were processed as previously described.

### Human tissue RNA sequencing

We accessed human brain tissue RNA sequencing performed by Target ALS and the New York Genome Center (http://www.targetals.org/research/resources-for-scientists/resource-genomic-data-sets/). Sixteen cases of control frontal cortex, eight *C9orf72* ALS/FTD frontal cortex, 15 control motor cortex, 12 *C9orf72* ALS/FTD frontal cortex, four control occipital cortex, and five *C9orf72* ALS/FTD frontal cortex were evaluated for differential expression of microglia specific genes.

### RNA sequencing analysis

Fastq files were quality- and adapter-trimmed using cutadapt (version 1.14). Adapter-trimmed fastq files were then aligned to the human genome (hg38, gencode v29) using STAR (version 2.6.1d) with default options. RNA count matrices were pulled from aligned BAM files using featureCounts (version 1.6.4). All downstream statistical analysis was done in R (version 3.6.2) using raw count matrices from featureCounts as input. Low-expression genes were filtered such that genes with mean read counts <10 were removed from the analysis. Differential expression analysis was done using DESeq2 (version 1.26.0) using disease status or treatment regime as the model. Volcano plots were generated from DESeq2 output using EnhancedVolcano. Heatmaps were generated using heatmap from z-scores calculated from DESeq2 normalized gene counts. Tissue data from Target ALS was downloaded from the New York Genome Center as raw fastq files and pushed through an identical analysis pipeline as data generated in our lab.

### Repeat primed PCR to detect the presence of C9 HRE in iPSC and iPSC-MG

We followed previously established protocols (Renton et al., 2011a) to determine the presence of the hexanucleotide repeat expansion (G_4_C_2_: >30) in *C9orf72* of iPSC and iPSC-MG (Supplementary Table S2).

### Real-time quantitative RT-PCR

RNA was isolated using Qiagen RNeasy Micro Kit (Cat #74004) according to the manufacturer's instructions. RNA was reverse transcribed to cDNA with oligo(dT) with the Promega Reverse Transcriptase System (Cat # A3500) and analyzed using SYBR Green Master Mix (Applied Biosystems). *C9orf72* (Forward- 5′- CAGTGATGTCGACTCTTTG−3′ and Reverse- 5′ AGTAGCTGCTAATAAAGGTGATTTG−3′) expression was normalized to RPL13A (Forward- 5′ CCTGGAGGAGAAGAGGAAAGAGA-3′ and Reverse- 5′ TTGAGGACCTCTGTGTATTTGTCAA-3′) or B2M (Forward- 5′ TGCTGTCTCCATGTTTGATGTATCT-3′ and Reverse- 5′ TCTCTGCTCCCCACCTCTAAGT-3′).

### Western blotting

Microglia cell pellets were homogenized in RIPA lysis and extraction buffer (Thermo Scientific, Cat #89900), supplemented with protease inhibitor cocktail (complete, Roche) and phosphatase inhibitor cocktail (PhosSTOP, Roche). Protein concentration was determined by a BCA assay kit (Thermo Fisher, Cat# 23225). Cell lysates were separated on 4–20% protean TGX precast gels (Biorad, Cat # #4561096) and blotted onto nitrocellulose membranes (Biorad, Cat # 1704159). Membranes were blocked for 60 min with Odyssey blocking buffer (PBS, Li-Cor, Cat #927-40000) and incubated overnight at 4°C with anti-C9orf72 (GeneTex Cat #. GTX634482, 1:1000) and anti-actin (Sigma-Aldrich A5441, 1:5000) antibodies. We used bone marrow-derived macrophages (BMDM) from a *C9orf72* knockout mouse (O'Rourke et al., 2016) to validate the antibody used for the Western blot analysis. After washing, membranes were incubated for 60 min with IRDye fluorescent secondary antibodies (Li-Cor). After washing, blots were subsequently analyzed with a Li-COR imaging system (Odyssey CLx). The Western blots were imaged and analyzed using the LICOR Odyssey imaging system which allows visualizing both faint and strong signals in a single image using increased sensitivity and no image saturation. Overexposure of high abundance proteins such as actin can mask the sample-to-sample variation of low-abundant proteins such as C9orf72, which can be overcome by using digital systems like the Licor Odyssey imaging platform.

### Repeat hybridization chain reaction (R-HCR) to detect intranuclear repeat RNA foci

We followed previously published methods (Glineburg et al. 2021). All main reagents and probes were purchased from Molecular Instrument, Inc. The negative control probe lacked any binding region with the tail ends of the initiator probe that binds to the hairpins. In brief, on DIV 30 of microglia differentiation, iPSC-MGs were plated onto eight chambers glass bottom slides (Ibidi #80827) coated with human fibronectin (sigma # F0895; 1:40) at a cell density of 125 000–150 000 cells per chamber. *Fixation step:* One hour after plating, iPSC-MG were washed once with 1x PBS and then fixed with 4% PFA for 10 min at room temperature. We further fix cells in 70% cold ethanol overnight at 4°C. *Initiation/Hybridization step:* Next day, 70% cold ethanol was removed and 250 μl per well of preheated hybridization buffer was added to cells and incubated at 45°C for 30 min. The hybridization buffer was then replaced with 125 μl of pre-warmed initiation probe and incubated at 45°C overnight (12–16 h). Approximately 4 nM C9 (CCCCGG)_6_ and negative scramble initiator probes solutions were prepared in a 45°C pre-warmed hybridization buffer. *Amplification step:* Six hours before starting the amplification stage, the R-HCR probe washing buffer was thawed and warmed up at 45°C. Cells were then washed 4x with pre-warmed (45°C) HCR probe wash buffer for 5 min at 45°C. Then washed 2x for 5 min with 5x SSC-T (5 X SSC, 0.1 % Tween20) at room temperature. Cells were then left in 5x SSC-T in a humid chamber at room temperature until the amplification stage was started. Approximately 1 h before the start of the amplification stage, we thawed the amplification buffer and hairpin probes B1H1 and B1H2 Alexa Fluor-546 to room temperature. Separately, hairpin B1H1 and hairpin B1H2 were prepared by snap cooling to the appropriate volume of the 3 μM stock. Approximately 15 nM of each hairpin probe was used. Each hairpin was heated up at 95°C for 90 s, placed on ice, and then left in a dark drawer at room temperature (no ice) for 30 min. We combined the snap-cooled B1H1 hairpins and snap-cooled B1H2 hairpins in the appropriate volume of amplification buffer at room temperature. The pre-amplification solution was removed and 125 μl per well of the hairpin solution was added to each chamber. iPSC-MGs were incubated cells with hairpin probes in the dark at room temperature overnight (12–16 h). The next day, cells were washed x5 for 5 min each in 5x SSC-T at room temperature. Then, incubated with DAPI (made in 5x SSC-T) for 10 min at room temperature. Wash cells 2x for 7 min each in 5x SSC-T at room temperature. Mount with Ibidi mounting medium (Ibidi #50001). IPSC-MGs were then visualized and imaged using a Zeiss LSM800 laser scanning confocal microscope. Using Imaris Software from Bitplane, we quantified the percentage of iPSC-MG presenting HRE-associated nuclear foci and the total number of foci per cell.

### Immunoassay analysis of poly-(GP)

Levels of poly-(GP) in cell lysates were measured in a blinded fashion using a Meso Scale Discovery (MSD) immunoassay and an MSD QUICKPLEX SQ120 instrument. A purified mouse monoclonal poly-(GP) antibody was used as both the capture and detection antibody (TALS 828.179, Target ALS Foundation). The capture antibody was biotinylated and used to coat 96-well MSD Small Spot Streptavidin plates, whereas the detection antibody was tagged with an electrochemiluminescent label (MSD GOLD SULFO-TAG). Lysates were diluted to the same protein concentration, and each sample was tested in duplicate. For each well, the intensity of emitted light, which is reflective of poly-(GP) levels and presented as arbitrary units, was acquired upon electrochemical stimulation of the plates.

### IPSC-MG cytokine assay

On DIV 29, iPSC-MGs were plated onto four chamber glass slides coated with human fibronectin (sigma # F0895; 1:40) at a cell density of 250 000 cells per chamber. On DIV 30, control and disease iPSC-MGs were treated with LPS (100 ng/mL) for 6 h, based on previous studies (Balez et al., 2016; Muffat et al., 2016). The conditioned media was collected and analyzed for selective cytokine/chemokine profile using the U-PLEX Biomarker Group 1 (Human; Mesoscale #K15067L) Multiplex Assay per manufacturer's protocol.

### LPS and Aβ (1-40) stimulation of iPSC-MG

On DIV 28–30, iPSC-MG from four *C9orf72* ALS/FTD patient lines and five control lines were plated onto four chamber glass slides as described above. For *LPS stimulation:* One hour after plating, iPSC-MGs were treated for 6 h with LPS (100 ng/mL). All iPSC-MG were then collected and sent for RNA sequencing analysis. For *A*β *(1-40) stimulation*, iPSC-MGs were treated for 2 h with vehicle (DMSO) or 1 μM Aβ (1–40) TAMRA (human Aβ, AnaSpec # AS-60488; Paolicelli et al., 2017) prepared in microglia basal media with growth factors. All iPSC-MGs were collected at 2 h and sent for RNA sequencing analysis.

### Phagocytosis of Aβ (1-40) TAMRA protein by iPSC-MG

On DIV 30 of microglia differentiation, iPSC-MGs were plated onto four chamber glass slides coated with human fibronectin (sigma # F0895; 1:40) at a cell density of 250 000 cells per chamber. One hour after plating, iPSC-MGs were treated for 5 min with vehicle (DMSO) or 1 μM of fluorescently labeled Aβ (1–40) TAMRA (human Aβ, AnaSpec # AS-60488; Paolicelli et al., 2017) prepared in microglia basal media with growth factors. All iPSC-MGs were washed at 5 min with microglia basal media supplemented with growth factors. IPSC-MGs were then fixed after 5 min, 30 min, and 1 h and immunostained for TREM2 as described above. Only 5 min and 1 h time points were quantified. Cells were imaged using a Zeiss LSM800 confocal microscope. Using Imaris Software from Bitplane, we determine the cell volume and percentage of the microglia surface area covered by Aβ (1-40) TAMRA at different time points.

### Engulfment of human brain synaptoneurosomes by iPSC-MG

Fluorescently labeled pHrodo (a pH-sensitive dye that fluoresces only in acidic compartments) human brain synaptoneurosomes (hSN-rodo) were generated as described previously (Hesse et al., 2019; Tzioras et al., 2019). On DIV 30 of the microglia differentiation, iPSC-MGs were plated onto eight chamber glass slides that were coated with human fibronectin (sigma # F0895; 1:40) at a cell density of 100 000 cells per chamber. One hour after plating, we labeled live microglia with the nuclear marker Hoechst 33342 (Thermo Fisher # H3570). Then, control and *C9orf72* iPSC-MGs were treated with 1:100 dilution of 4 mg/mL hSN-rodo in the presence or absence of 10 μM cytochalasin-D (inhibitor of actin polymerization). Confocal live cell imaging of iPSC-MGs was done using a 20X objective of a Zeiss 800 confocal microscope. IPSC-MGs were imaged every 10 min for up to 6 h for initial phagocytosis studies to determine the percentage of cells that exhibit phagocytic activity. For all other sets of experiments, iPSC-MGs were imaged every 10 min for up to 2 h. All lines had three technical replicates for the hSN-rodo treatment. Six images were taken per well per line for a total of 18 images every 10 min. All images were analyzed using the Imaris Software from Bitplane. To determine the percentage of iPSC-MGs engulfing hSN-rodo, the spots module was used to count the total number of iPSC-MGs based on the Hoechst marker and the total number of phagocytic iPSC-MGs per image. Additionally, we quantified the fluorescence mean intensity of the cargo hSN-rodo per phagocytic cell at the 2 h time point, where more than 60% of the iPSC-MGs were engulfing hSN-rodo. Cytochalasin-D was used as a negative control.

### Confocal microscopy and bright-field imaging

All immunostained iPSC-MGs were visualized and imaged using a Zeiss LSM800 laser scanning confocal microscope. Per staining, all images were taken with the same settings for parallel cultures. For all iPSC-MG immunostainings and Aβ (1-40) TAMRA phagocytic activity assay, a plan Apochromat 63x oil immersion objective was used; Z-stacks were generated with 1024 x 1024 image size, 0.5x XY scan zoom, and 1 μm scaling. For some immunostainings, differential interference contrast (DIC) was used to highlight the iPSC-MG surface area. For live cell imaging of iPSC-MG engulfing synaptoneurosomes, confocal microscopy with differential interference contrast was used. Tiled images were captured using a 20X objective with a 1.0x XY scan zoom and 0.624 μm x 0.624μm scaling. Bright-field images of the iPSC-MG cultures were taken using a Zeiss AxioVert.A1 microscope and a resolve HD Ludesco camera.

### Imaging analysis using Imaris Software from Bitplane

All images were processed and analyzed using the Imaris Software 9.5.1 and 9.6 from Bitplane. To obtain volume, area, mean intensity, and sum intensity per cell for a large number of samples, we assigned a randomized color identification per cell followed by the use of the ImarisVantage module to extract multiple numerical values from the created three-dimensional structures. For *iPSC-MG marker characterization*, the spots module was used to count the total number of cells positive for a specific microglia marker per image while using the DAPI channel as a reference. To calculate the *TDP-43 and ADAR-2 nucleocytoplasmic ratio (N/C ratio)*, we used the surface module to generate iPSC-MG microglia 3-dimensional cellular (based on membrane staining with wheat germ agglutinin) and nuclear surfaces (based on DAPI). The sum intensity of TDP-43 or ADAR-2 was acquired for the nucleus and cytoplasm as well as the volume for each cellular compartment. The following formula was used: (Cell Sum Intensity – Nucleus Sum Intensity) = Cytoplasm Sum Intensity; (Cell Volume – Nucleus Volume) = Cytoplasm Volume. *N/C ratio* = (Nucleus Sum Intensity/Nucleus volume)/(Cytoplasm Sum Intensity/Cytoplasm volume). Data from each cell were acquired by assigning randomized color identification followed by the use of ImarisVantage. For the *A*β *(1-40) TAMRA phagocytic activity*, we used *A*β *(1-40) TAMRA* fluorescence signal and chose an ideal threshold to create the *A*β surfaces inside the IPSC-MGs. TREM2 staining was used to generate the cell surface structures. An algorithm was also generated and used in all iPSC-MG parallel cultures. Randomized color identification and ImarisVantage was also used to extract all data like previous datasets. For *hSN-rodo live cell imaging over time*, the spots module was used to count the total number of cells over time. Phagocytic cells were manually identified based on the hSN-rodo signal inside iPSC-MG. We set a threshold between 10,000 and 12,000 units above the grayscale as the positive signal indicative of engulfment and lysosomal internalization. At the 2 h time point, the cell surface area of phagocytic cells was manually outlined using the Imaris Software manual creation tool and the hSN-rodo mean intensity value was obtained per cell. For *iPSC-MGs EEA1 and Lamp1 analysis*, based on the staining pattern, we manually choose an optimal threshold for each protein marker to generate the 3D surfaces. We then stored the surface algorithms and used them in all iPSC-MG parallel cultures. Randomized color identification and ImarisVantage were then used to extract the cell volume and mean intensity of both markers per cell.

### Statistical analysis

Statistical analysis for RNA sequencing was done in R (version 3.6.2) and detailed above. All other statistical analyses were performed using Graphpad Prism 7 and 8. For the comparison of the two groups, we used a two-tailed Student's *t*-test. Two-way ANOVA was used to analyze the difference between the means of more than two groups. One-way ANOVA with a *post-hoc* Dunnett's correction was performed to compare every cell line mean to the average of the control group. Two-tailed Mann–Whitney test was performed for the poly-(GP) ELISA assay. All statistical significance was ranked as the following: ^*^p ≤ 0.05; ^**^p ≤ 0.01; ^***^p ≤ 0.001; ^****^p ≤ 0.0001; and *p* > 0.05 not significant. All other statistical details and exact *p*-values are reported in each Figure legend.

## Data availability statement

All RNA sequencing data generated for this article is publicly available via GEO accession number GSE233262. All other datasets, protocols and analysis supporting the conclusion of this article are available upon request to the corresponding author.

## Ethics statement

Written informed consent was obtained from the individual(s) for the publication of any potentially identifiable images or data included in this article.

## Author contributions

IL: designed experiments, differentiated iPSC-MG, performed and supervised experiments, performed quantitative confocal microscope image analysis, acquired and analyzed data, and wrote the manuscript. EA and RP: performed RNA sequencing analysis for iPSC-MG and human postmortem tissue. JL: differentiated and maintained iPSC-MG. LG, BR, and DB: acquired data and performed quantitative microscope image analysis. DL, LB, JS, TM, and TG: performed and analyzed experiments. MT, AL, and JR: performed experiments. SM and DB: performed confocal imaging. EH: RNA sequencing analysis. MS, CD, and MM: performed quantitative image analysis. CB: maintained and expanded iPSCs. F-BG, SA, JI, and STH: provided *C9orf72* iPSC lines. MB-J and AM: taught first-hand iPSC-MG protocol. TS-J: provided hSN-rodo. RB and RHB: provided suggestions and evaluation of our work. KVK-J: designed experiments and provided critical advice and evaluation of our work. RS: designed experiments, oversaw data analysis and interpretation, and participated in manuscript writing and editing. All authors contributed to the article and approved the submitted version.

## Supplementary material

Supplementary Figure 1 Expression of microglia protein markers and microglia-specific genes in healthy control and *C9orf72* ALS/FTD iPSC-derived microglia. **(A)** Percentage of DAPI-positive cells from mature healthy control (*n* = 3 lines, 1–2 differentiations per line) and *C9orf72* ALS/FTD (*n* = 3–5 lines, 1–2 differentiations per line) iPSC-MG positive for P2ry12, Cx3cr1, TREM2, and TMEM119 protein. **(B)** Dot plots showing the level of expression as Log2 (counts +1) of microglia-specific genes *spI1* (encodes for myeloid transcription factor PU.1), *P2RY12, CX3CR1, TREM2*, and *TMEM119* in healthy control and *C9orf72* ALS/FTD (Control, *n* = 4 lines, 1–2 differentiations per line; *C9orf72, n* = 7 lines, 1–2 differentiations per line).

Supplementary Figure 2 Differentially expressed transcripts in *C9orf72* ALS/FTD vs. healthy control iPSC MG full transcriptome and dysregulated microglial-enriched transcripts in *C9orf72* ALS/FTD frontal, motor, and occipital cortex. **(A)** Top differentially expressed transcripts in iPSC MG full transcriptome (unadjusted *p* < 0.005; log_2_ fold change (FC) ± 1). **(B)** Top microglial-enriched dysregulated transcripts in the frontal and motor cortex of *C9orf72* ALS/FTD patient tissue from bulk RNA sequencing. **(C)** Volcano plot of differentially expressed microglial-enriched genes (total of 881 from Gosselin et al., 2017) in *C9orf72* ALS/FTD occipital cortex (control, *n* = 4 lines; *C9orf72, n* = 5 lines) (unadjusted *p* < 0.05; log_2_ fold change (FC) ± 1). **(D)** Dysregulated transcripts in the occipital cortex of *C9orf72* ALS/FTD patient tissue from bulk RNA sequencing.

Supplementary Figure 3 Heatmap of the homeostatic, interferon, NF-kB, and activated response genes in iPSC-MG and human cortex. Expression of **(A, E)** homeostatic, **(B–F)** interferon, **(C–G)** NF-kB, and **(D–H)** activated response genes in iPSC-MGs (control, *n* = 4 cell lines with 1–2 differentiations each and *C9orf72* ALS/FTD, *n* = 7 cell lines with 1–2 differentiations each) and human cortex (frontal cortex control *n* = 16 and *C9orf72* ALS/FTD *n* = 8; motor cortex control *n* = 15, *C9orf72* ALS/FTD *n* = 12, occipital cortex control *n* = 4, and *C9orf72* ALS/FTD *n* = 5).

Supplementary Figure 4 *C9orf72* ALS/FTD iPSC-MG mono-cultures do not exhibit TDP-43 pathology or cytoplasmic mislocalization of ADAR2. **(A)** TDP-43 nuclear staining in control and *C9orf72* ALS/FTD iPSC-MG. IPSC-MG cell surface and the nuclear surface are outlined (white). Scale bar, 15 μm. **(B)** Control and *C9orf72* ALS/FTD iPSC-MG immunostained for anti-ADAR2. IPSC-MG nuclear and cell surface is outlined (white). Scale bar, 10 μm. **(C)** Quantification of TDP-43 nucleocytoplasmic ratio using the Imaris Software. No evidence of cytoplasmic accumulations in *C9orf72* ALS/FTD iPSC-MG mono-cultures (control, *n* = 5 lines; *C9orf72, n* = 5 lines, *n* = 60–84 cells per line; *p* = 0.85, Student's *t*-test). **(D)** Quantification of ADAR2 nucleocytoplasmic ratio. No evidence of nuclear ADAR2 mislocalization to the cytoplasm was observed in *C9orf72* ALS/FTD iPSC-MG mono-cultures (control, *n* = 5 lines; *C9orf72, n* = 5 lines, *n* = 100–115 cells per line; *p* = 0.39, Student's *t*-test).

Supplementary Figure 5 *C9orf72* ALS/FTD iPSC-MG response to LPS stimulation. Control IPSC-MGs (*n* = 4) and *C9orf72* ALS/FTD iPSC-MG (*n* = 3) were treated with LPS (100 ng/ml) for 6 h. Conditioned media samples were collected and measured by a U-plex Biomarker Group 1 (Human, Mesoscale) Multiplex Assay ELISA to obtain a cytokine/chemokine profile of iPSC-MG. Here, we present the concentration (pg/mL) of **(A)** IL1α, **(B)** IL1β, **(C)** IL6, and **(D)** TNFα per replicate within a sample. **(E)** Top differentially expressed genes in LPS-treated *C9orf72* ALS/FTD vs. healthy control iPSC-MGs.

Supplementary Figure 6 Top differentially expressed genes in LPS or Aβ (1–40) treated healthy control and *C9orf72* ALS/FTD iPSC-MGs. Mature control (*n* = 5) and *C9orf72* ALS/FTD (*n* = 4) iPSC-MG were treated for 6 h with LPS (100 ng/mL) or 2 h with 1 μM Aβ (1–40) TAMRA. All iPSC-MGs were collected after stimulation and sent for RNA sequencing analysis. Significantly dysregulated genes (log_2_ fold change (FC) ± 1, p-value <0.05) were observed in both LPS-treated healthy controls **(A)** and LPS-treated *C9orf72* ALS/FTD iPSC-MGs **(B)**. Minimal dysregulated genes were observed for all iPSC-MG treated with Aβ (1–40) TAMRA **(C, D)**. **(E)** List of the top differentially expressed genes in Aβ (1–40) TAMRA treated *C9orf72* ALS/FTD vs. healthy control iPSC-MG.

Supplementary Figure 7 *C9orf72* ALS/FTD iPSC-MG engulf and degrade human pHrodo synaptoneurosomes containing PSD-95 and synaptophysin 38. **(A)** Human brain pHrodo synaptoneurosomes (hSN-rodo) contain PSD95 and Synaptophysin 38 proteins from pre- and post-synaptic compartments. Western blot analysis for total homogenate (TH) and synaptoneurosome preparations (SN). Four different SN preparations (TH1–TH4 and SN1–SN4) from four different control brains were frozen samples. Preparations SN1, SN3, and SN4 had successful enrichment of post-synaptic density 95 (PSD95–95 kDa) and synaptophysin (Synaptophysin 38kDa) compared to the total homogenate. In addition, low levels of nuclear marker histone 3 were observed in preparations SN1, SN3, and SN4. SN1 (white arrow) was the preparation used for the present study. **(B)** PSD95, synaptophysin 38, and Histone 3 quantification for TH and SN preparations 1, 3, and 4. A significant decrease in Histone 3 levels is observed in all SN preparations, p-value =0.02, Student's t-test. **(C)** Selected representative images of the time-lapse live cell imaging of *C9orf72* ALS/FTD iPSC-MG engulfing and degrading human synaptoneurosomes pHrodo (hSN-rodo). IPSC-MGs were labeled with the live nuclear marker Hoechst (blue) to identify individual cells followed by treatment with hSN-rodo (red). Fluorescent live cell imaging with differential interference contrast microscopy was performed over a 2-h time frame with images taken every 10 min. Here, we present selected images during the T0–T2 h time point where more than 60% of iPSC-MGs engulf synapses. HSN-rodo is engulfed rapidly (white arrows highlight several phagocytic iPSC-MGs) and an increase in hSN-rodo intensity is observed in individual cells indicating the uptake of hSN-rodo into acidic intracellular compartments.

Table S1 Demographics for *C9orf72* ALS/FTD patient and control iPSCs.

Table S2 Presence of *C9orf72* HRE in iPSC and iPSC-MG cells.

Table S3 List of iPSC lines used for specific experiments.

Table S4 Postmortem tissue sample IDs were used for bulk RNA-seq analysis from Target ALS collection.

Table S5 List of antibodies used to stain iPSC-MG cells.

Video S1 Time-lapse live cell imaging of *C9orf72* ALS/FTD iPSC-MG engulfing and degrading human synaptoneurosomes pHrodo (hSN-rodo). Here, we present selected time-lapse live cell imaging of *C9orf72* ALS/FTD iPSC-MG engulfing hSN-rodo. Fluorescent live cell imaging with differential interference contrast microscopy was performed over a 6 h and 2 h time frame with images taken every 10 min. IPSC-MGs were identified using the nuclear marker Hoechst (blue) followed by treatment with hSN-rodo (red). The increase in hSN-rodo intensity is observed in individual cells indicative of the uptake of hSN-rodo into acidic intracellular compartments for degradation.
