# Supplementary figures and images for: Moderate intrinsic phenotypic alterations in C9orf72 ALS/FTD iPSC-microglia despite the presence of C9orf72 pathological features
Source: Front Cell Neurosci. 2023 Jun 6;17:1179796. doi: 10.3389/fncel.2023.1179796 (PMC10279871; doi:10.3389/fncel.2023.1179796)

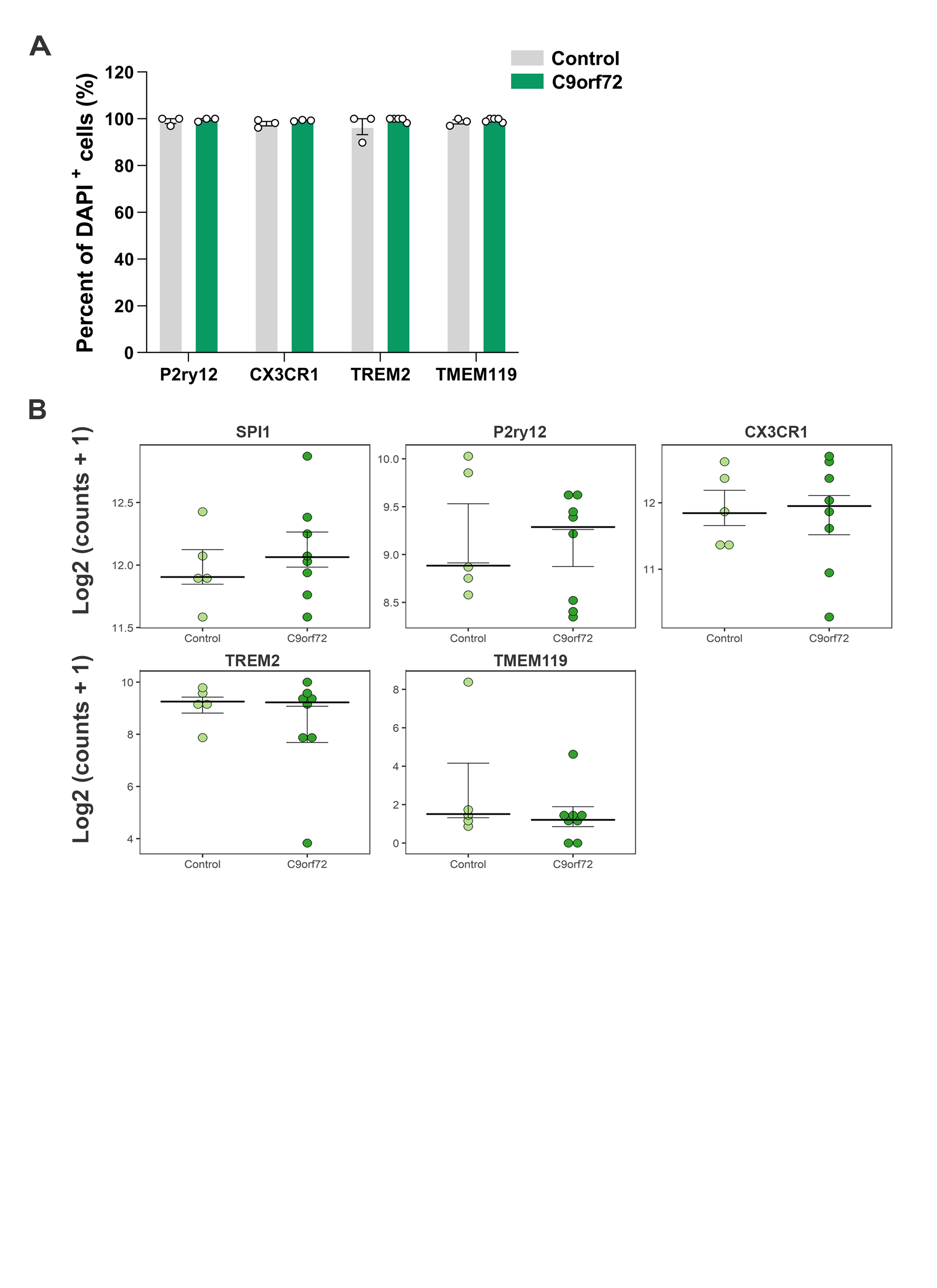

Supplement: Supplementary Figure 1 — Expression of microglia protein markers and microglia-specific genes in healthy control and C9orf72 ALS/FTD iPSC-derived microglia. (A) Percentage of DAPI-positive cells from mature healthy control (n = 3 lines, 1–2 differentiations per line) and C9orf72 ALS/FTD (n = 3–5 lines, 1–2 differentiations per line) iPSC-MG positive for P2ry12, Cx3cr1, TREM2, and TMEM119 protein. (B) Dot plots showing the level of expression as Log2 (counts +1) of microglia-specific genes spI1 (encodes for myeloid transcription factor PU.1), P2RY12, CX3CR1, TREM2, and TMEM119 in healthy control and C9orf72 ALS/FTD (Control, n = 4 lines, 1–2 differentiations per line; C9orf72, n = 7 lines, 1–2 differentiations per line). [file Image_1.TIFF]

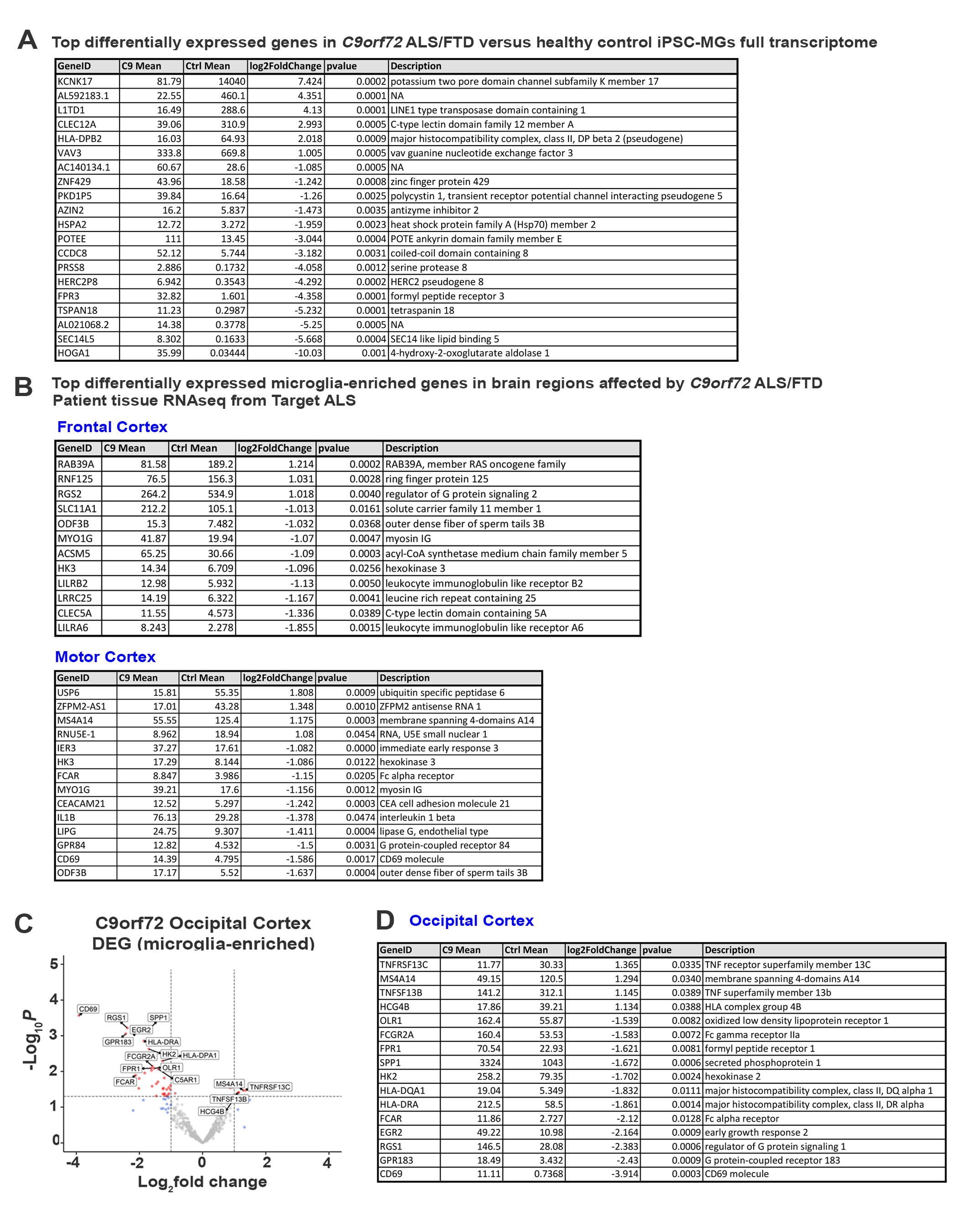

Supplement: Supplementary Figure 2 — Differentially expressed transcripts in C9orf72 ALS/FTD vs. healthy control iPSC MG full transcriptome and dysregulated microglial-enriched transcripts in C9orf72 ALS/FTD frontal, motor, and occipital cortex. (A) Top differentially expressed transcripts in iPSC MG full transcriptome (unadjusted p < 0.005; log2 fold change (FC) ± 1). (B) Top microglial-enriched dysregulated transcripts in the frontal and motor cortex of C9orf72 ALS/FTD patient tissue from bulk RNA sequencing. (C) Volcano plot of differentially expressed microglial-enriched genes (total of 881 from Gosselin et al., 2017) in C9orf72 ALS/FTD occipital cortex (control, n = 4 lines; C9orf72, n = 5 lines) (unadjusted p < 0.05; log2 fold change (FC) ± 1). (D) Dysregulated transcripts in the occipital cortex of C9orf72 ALS/FTD patient tissue from bulk RNA sequencing. [file Image_2.TIFF]

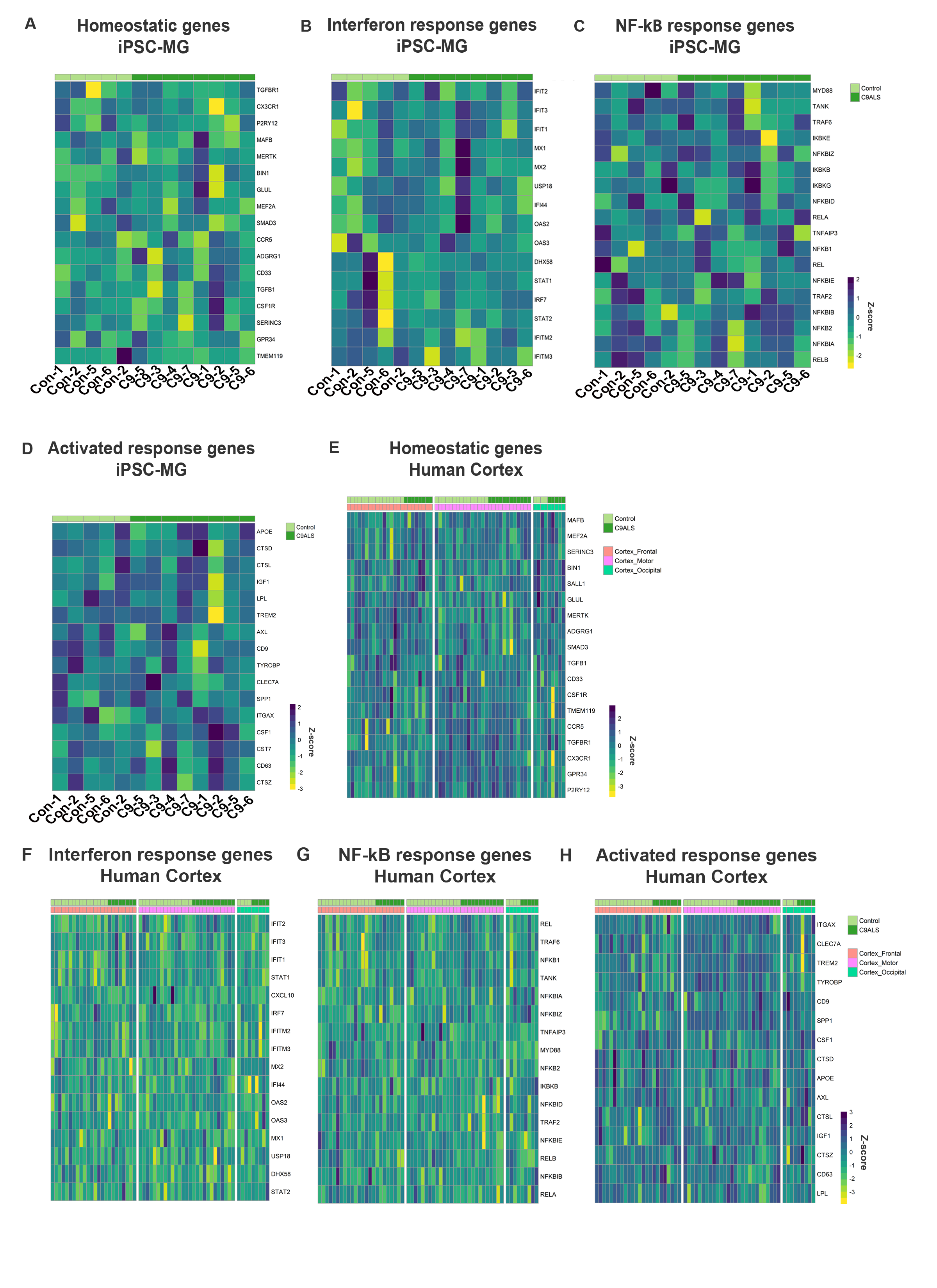

Supplement: Supplementary Figure 3 — Heatmap of the homeostatic, interferon, NF-kB, and activated response genes in iPSC-MG and human cortex. Expression of (A, E) homeostatic, (B–F) interferon, (C–G) NF-kB, and (D–H) activated response genes in iPSC-MGs (control, n = 4 cell lines with 1–2 differentiations each and C9orf72 ALS/FTD, n = 7 cell lines with 1–2 differentiations each) and human cortex (frontal cortex control n = 16 and C9orf72 ALS/FTD n = 8; motor cortex control n = 15, C9orf72 ALS/FTD n = 12, occipital cortex control n = 4, and C9orf72 ALS/FTD n = 5). [file Image_3.TIFF]

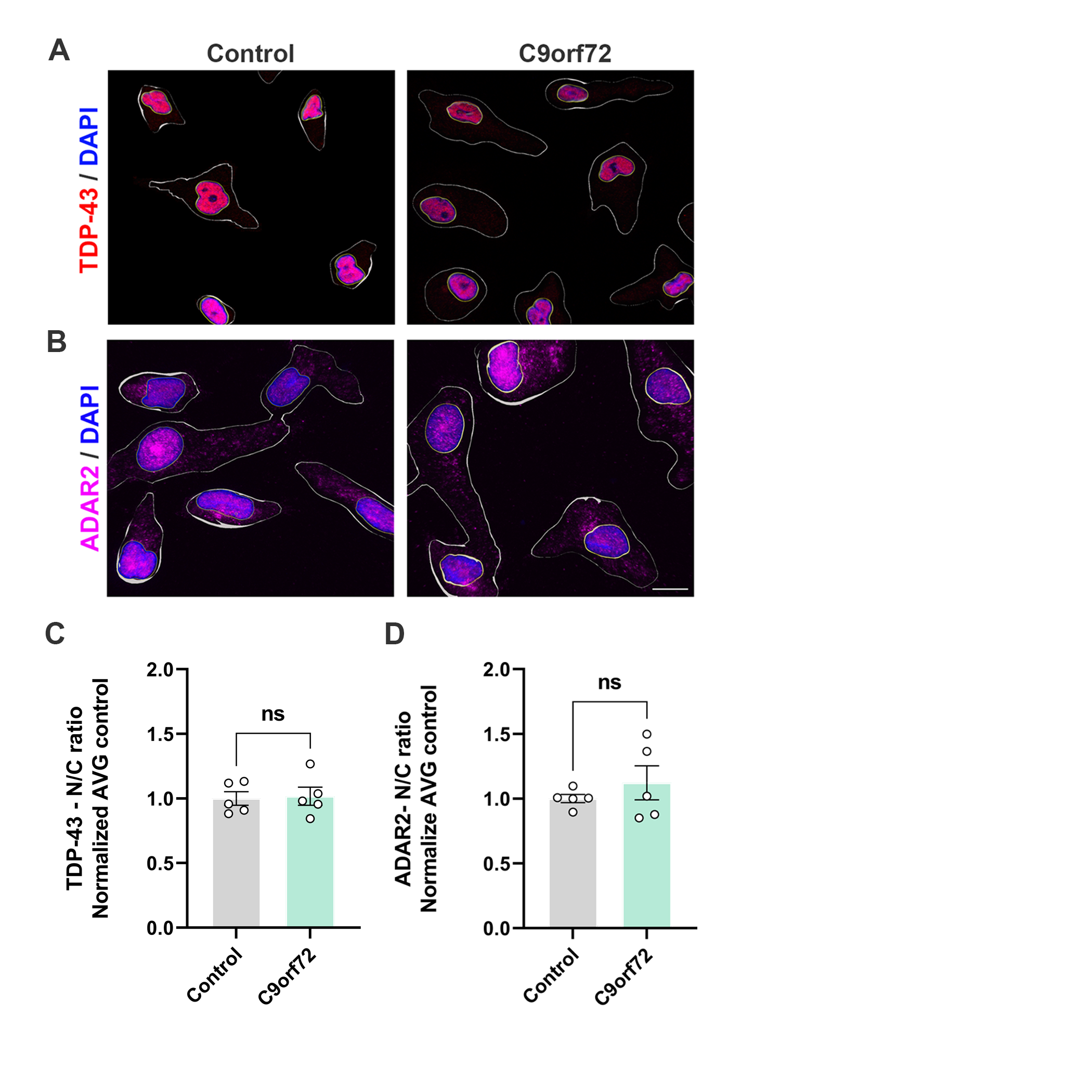

Supplement: Supplementary Figure 4 — C9orf72 ALS/FTD iPSC-MG mono-cultures do not exhibit TDP-43 pathology or cytoplasmic mislocalization of ADAR2. (A) TDP-43 nuclear staining in control and C9orf72 ALS/FTD iPSC-MG. IPSC-MG cell surface and the nuclear surface are outlined (white). Scale bar, 15 μm. (B) Control and C9orf72 ALS/FTD iPSC-MG immunostained for anti-ADAR2. IPSC-MG nuclear and cell surface is outlined (white). Scale bar, 10 μm. (C) Quantification of TDP-43 nucleocytoplasmic ratio using the Imaris Software. No evidence of cytoplasmic accumulations in C9orf72 ALS/FTD iPSC-MG mono-cultures (control, n = 5 lines; C9orf72, n = 5 lines, n = 60–84 cells per line; p = 0.85, Student's t-test). (D) Quantification of ADAR2 nucleocytoplasmic ratio. No evidence of nuclear ADAR2 mislocalization to the cytoplasm was observed in C9orf72 ALS/FTD iPSC-MG mono-cultures (control, n = 5 lines; C9orf72, n = 5 lines, n = 100–115 cells per line; p = 0.39, Student's t-test). [file Image_4.TIFF]

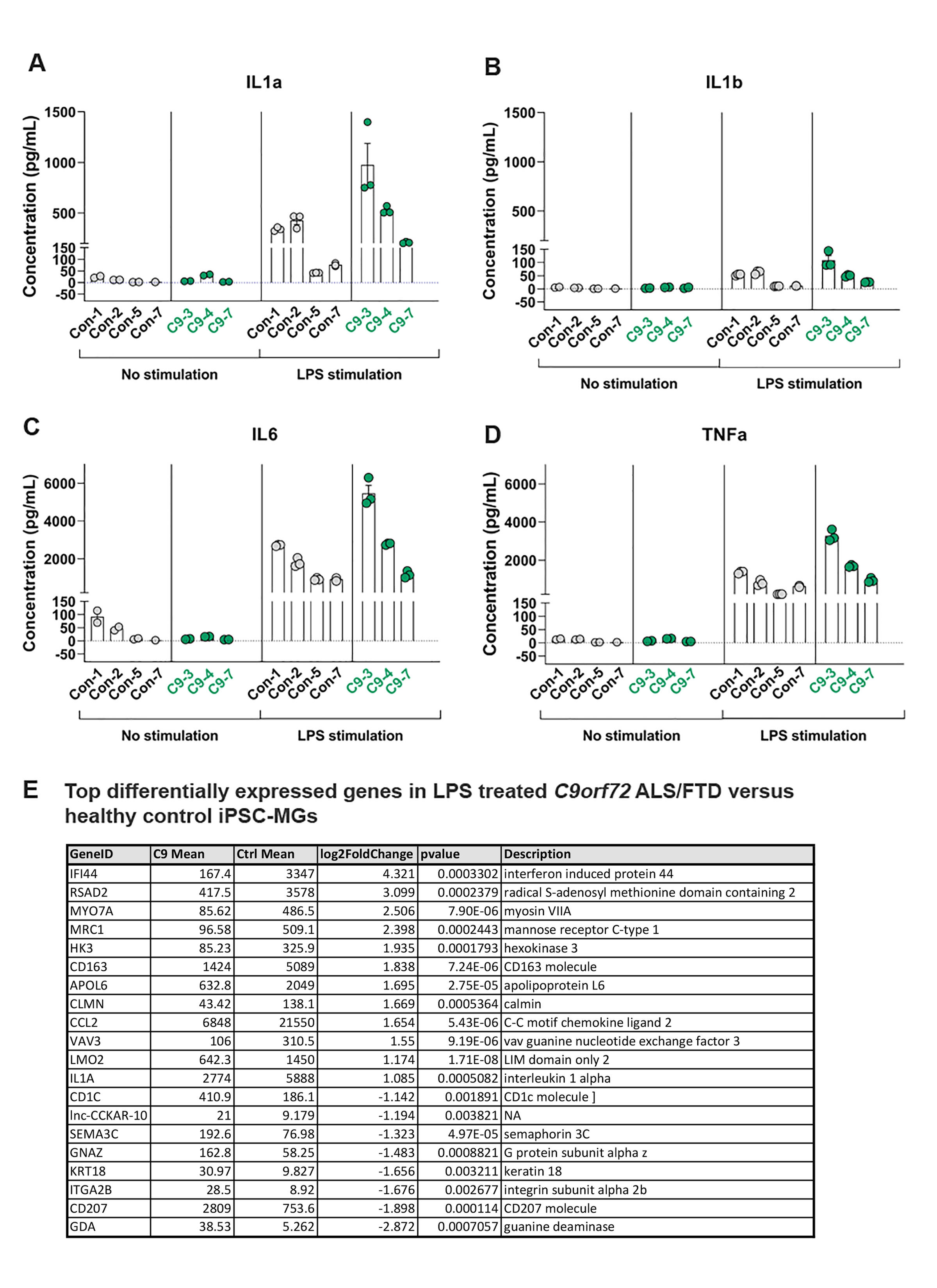

Supplement: Supplementary Figure 5 — C9orf72 ALS/FTD iPSC-MG response to LPS stimulation. Control IPSC-MGs (n = 4) and C9orf72 ALS/FTD iPSC-MG (n = 3) were treated with LPS (100 ng/ml) for 6 h. Conditioned media samples were collected and measured by a U-plex Biomarker Group 1 (Human, Mesoscale) Multiplex Assay ELISA to obtain a cytokine/chemokine profile of iPSC-MG. Here, we present the concentration (pg/mL) of (A) IL1α, (B) IL1β, (C) IL6, and (D) TNFα per replicate within a sample. (E) Top differentially expressed genes in LPS-treated C9orf72 ALS/FTD vs. healthy control iPSC-MGs. [file Image_5.TIFF]

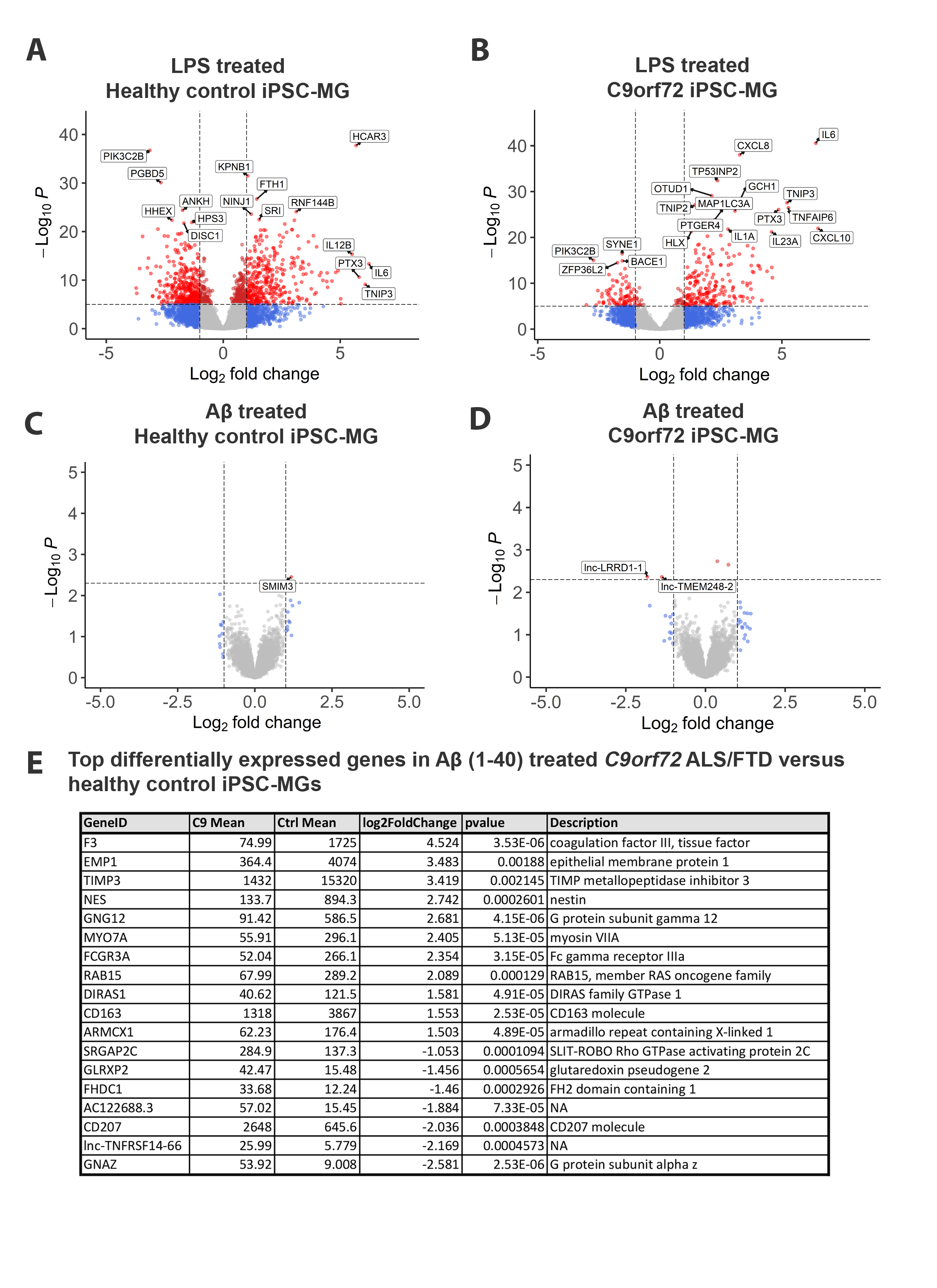

Supplement: Supplementary Figure 6 — Top differentially expressed genes in LPS or Aβ (1–40) treated healthy control and C9orf72 ALS/FTD iPSC-MGs. Mature control (n = 5) and C9orf72 ALS/FTD (n = 4) iPSC-MG were treated for 6 h with LPS (100 ng/mL) or 2 h with 1 μM Aβ (1–40) TAMRA. All iPSC-MGs were collected after stimulation and sent for RNA sequencing analysis. Significantly dysregulated genes (log2 fold change (FC) ± 1, p-value <0.05) were observed in both LPS-treated healthy controls (A) and LPS-treated C9orf72 ALS/FTD iPSC-MGs (B). Minimal dysregulated genes were observed for all iPSC-MG treated with Aβ (1–40) TAMRA (C, D). (E) List of the top differentially expressed genes in Aβ (1–40) TAMRA treated C9orf72 ALS/FTD vs. healthy control iPSC-MG. [file Image_6.TIFF]

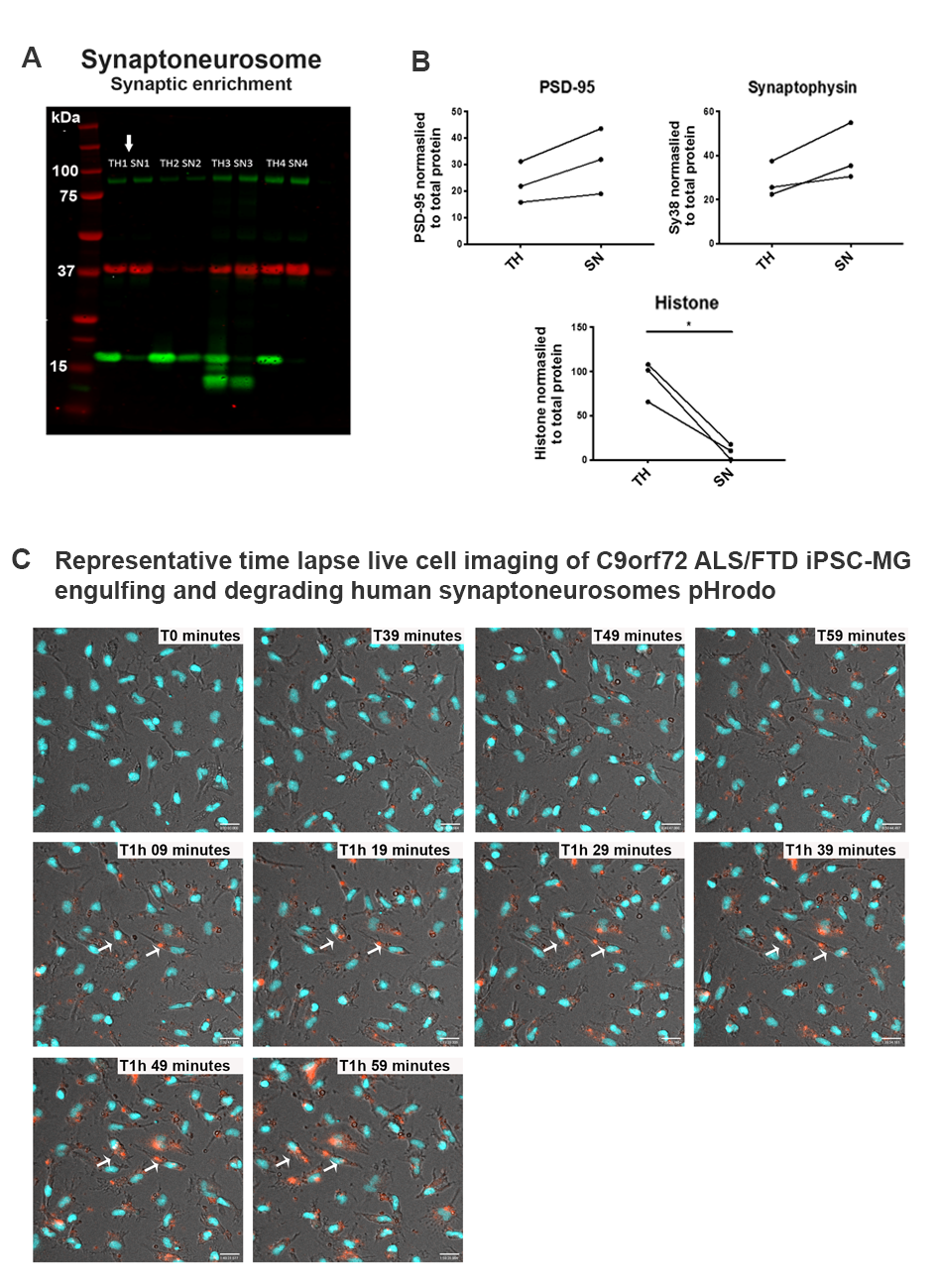

Supplement: Supplementary Figure 7 — C9orf72 ALS/FTD iPSC-MG engulf and degrade human pHrodo synaptoneurosomes containing PSD-95 and synaptophysin 38. (A) Human brain pHrodo synaptoneurosomes (hSN-rodo) contain PSD95 and Synaptophysin 38 proteins from pre- and post-synaptic compartments. Western blot analysis for total homogenate (TH) and synaptoneurosome preparations (SN). Four different SN preparations (TH1–TH4 and SN1–SN4) from four different control brains were frozen samples. Preparations SN1, SN3, and SN4 had successful enrichment of post-synaptic density 95 (PSD95–95 kDa) and synaptophysin (Synaptophysin 38kDa) compared to the total homogenate. In addition, low levels of nuclear marker histone 3 were observed in preparations SN1, SN3, and SN4. SN1 (white arrow) was the preparation used for the present study. (B) PSD95, synaptophysin 38, and Histone 3 quantification for TH and SN preparations 1, 3, and 4. A significant decrease in Histone 3 levels is observed in all SN preparations, p-value =0.02, Student's t-test. (C) Selected representative images of the time-lapse live cell imaging of C9orf72 ALS/FTD iPSC-MG engulfing and degrading human synaptoneurosomes pHrodo (hSN-rodo). IPSC-MGs were labeled with the live nuclear marker Hoechst (blue) to identify individual cells followed by treatment with hSN-rodo (red). Fluorescent live cell imaging with differential interference contrast microscopy was performed over a 2-h time frame with images taken every 10 min. Here, we present selected images during the T0–T2 h time point where more than 60% of iPSC-MGs engulf synapses. HSN-rodo is engulfed rapidly (white arrows highlight several phagocytic iPSC-MGs) and an increase in hSN-rodo intensity is observed in individual cells indicating the uptake of hSN-rodo into acidic intracellular compartments. [file Image_7.TIFF]
