# Supplementary material for: Moderate intrinsic phenotypic alterations in C9orf72 ALS/FTD iPSC-microglia despite the presence of C9orf72 pathological features
Source: Front Cell Neurosci. 2023 Jun 6;17:1179796. doi: 10.3389/fncel.2023.1179796 (PMC10279871; doi:10.3389/fncel.2023.1179796)
Supplement: Table S1 — Demographics for C9orf72 ALS/FTD patient and control iPSCs. [file Table_1.docx]

**Table S2. Presence of *C9orf72* HRE in iPSC and iPSC-MG cells**

**Supplemental Tables: Tables S1-S5**

**Table S1. Demographics for *C9orf72* ALS/FTD patient and healthy control iPSCs**

**Occipital Cortex**

**Frontal Cortex**

**Motor Cortex**

**Table S4. Postmortem tissue sample IDs used for bulk RNAseq analysis from Target ALS**

**collection**

**Table S3. List of iPSC lines used for specific experiments**

**Table S5. List of antibodies used to stained iPSC-MG cells**
